# Nuclear Magnetic Resonance Metabolomics with Double Pulsed-Field-Gradient Echo and Automatized Solvent Suppression Spectroscopy for Multivariate Data Matrix Applied in Novel Wine and Juice Discriminant Analysis

**DOI:** 10.3390/molecules26144146

**Published:** 2021-07-07

**Authors:** José Enrique Herbert-Pucheta, José Daniel Lozada-Ramírez, Ana E. Ortega-Regules, Luis Ricardo Hernández, Cecilia Anaya de Parrodi

**Affiliations:** 1 Consejo Nacional de Ciencia y Tecnología-Laboratorio Nacional de Investigación y Servicio Agroalimentario y Forestal, Universidad Autónoma Chapingo, Carretera México-Texcoco km 38.5, Chapingo, Estado de México 56230, Mexico; jherbertp@ipn.mx; 2 Departamento de Química Orgánica, Escuela Nacional de Ciencias Biológicas, Instituto Politécnico Nacional, Prolongación de Carpio y Plan de Ayala s/n, Colonia Santo Tomás, Ciudad de México 11340, Mexico; 3 Departamento de Ciencias Químico Biológicas, Universidad de las Américas Puebla, San Andrés Cholula 72810, Mexico; jose.lozada@udlap.mx; 4 Departamento de Ciencias de la Salud, Universidad de las Américas Puebla, San Andrés Cholula 72810, Mexico; ana.ortega@udlap.mx

**Keywords:** ^1^H-NMR, multivariate statistical analysis, wine, juices, NMR pulse sequence, Cabernet Sauvignon, *Candida zemplinina*, *Saccharomyces Bayanus ex uvarum*

## Abstract

The quality of foods has led researchers to use various analytical methods to determine the amounts of principal food constituents; some of them are the NMR techniques with a multivariate statistical analysis (NMR-MSA). The present work introduces a set of NMR-MSA novelties. First, the use of a double pulsed-field-gradient echo (DPFGE) experiment with a refocusing band-selective uniform response pure-phase selective pulse for the selective excitation of a 5–10-ppm range of wine samples reveals novel broad ^1^H resonances. Second, an NMR-MSA foodomics approach to discriminate between wine samples produced from the same Cabernet Sauvignon variety fermented with different yeast strains proposed for large-scale alcohol reductions. Third a comparative study between a nonsupervised Principal Component Analysis (PCA), supervised standard partial (PLS-DA), and sparse (sPLS-DA) least squares discriminant analysis, as well as orthogonal projections to a latent structures discriminant analysis (OPLS-DA), for obtaining holistic fingerprints. The MSA discriminated between different Cabernet Sauvignon fermentation schemes and juice varieties (apple, apricot, and orange) or juice authentications (puree, nectar, concentrated, and commercial juice fruit drinks). The new pulse sequence DPFGE demonstrated an enhanced sensitivity in the aromatic zone of wine samples, allowing a better application of different unsupervised and supervised multivariate statistical analysis approaches.

## 1. Introduction

Analytical tools to follow the composition, origin, and traceability of beverages have become a common practice to evaluate the quality and characteristics of foods as robust alternatives of handheld equipment for food safety inspectors or in-line equipment for food authentication analyses, currently carried out as rapid outside laboratory methods [1]. Nuclear Magnetic Resonance (NMR) spectroscopy technology evaluates juices, extracts, infusions, and alcoholic, and nonalcoholic beverages. It has identified possible counterfeit products by monitoring the presence of specific metabolites within a sample [2]. This technique has also identified possible adulterations in coffee [3,4], tea bags for herbal infusions [5,6,7,8], vodka [9,10], milk and dairy products [11,12,13], honey [9,14,15], beer [9,16], oil [7,17,18], wine [2,19,20,21,22], juice [2,23,24]; vinegar [2,9,25], tequila [9,25,26], rum [9], and whiskey [9], amongst others. Spectroscopic and spectrometric techniques combined, in some cases, with chromatographic methods are useful to assure the food quality to avoid adulterations and fraud and determine the geographical origin of the constituent ingredients, because consumers consider this data as one of the principal quality indicators [27]. Hyphenated analytical methods have evaluated the influence of the harvest season on the characteristics of wine [28], with NMR and LC-MS analyses tracking variations in the concentrations of wine compounds. The authors found thirty-one metabolites by UPLC-MS (20 phenolic compounds, 8 hydroxy acids, and 3 apocarotenoids). They also recognized the differences in the compositions of those compounds in wines produced during two different seasons (July and December) by proton Nuclear Magnetic Resonance Spectroscopy (^1^H-NMR). The results evaluated from Syrah and Chenin Blanc wines from Brazil evidenced the variability in primary and secondary metabolites due to seasonality, training systems, and types of rootstocks.

Despite the restricted Limits of Quantification (LOQ) and Detection (LOD), ^1^H-NMR has been a useful method for the geographical origin authentication of several food matrices, with clear advantages of being nondestructive, fast, reproducible, and reliable, compared to chromatography coupled with MS techniques [19,20,29,30,31]. The combination of high-reproducible, noninvasive, rapid, and simple-use proton Nuclear Magnetic Resonance Spectroscopy (^1^H-NMR) with Multivariate Statistical Analysis (MSA) for foodstuff metabolomics has emerged over the last decades for the implementation of models to trace the food quality, origin, manufacture, or authenticity [32].

NMR foodomics has proved its importance in evaluating possible adulteration in commercial juice blends [23]. The use of NMR and MSA analyses has established the concentration of different compounds to guarantee the origin of pure juices. The fingerprints generated by the metabolites present in juices reveal proton NMR signals in three well-defined regions: the region from 0.5 to 3.0 ppm, corresponding to protons of organic acids and amino acids; from 3.0 to 6.0 ppm, corresponding to the presence of carbohydrates; and from 6.0 to 8.5 ppm, corresponding to protons of phenolic and aromatic compounds. The use of NMR coupled to chemometrics generates valuable information capable of detecting corrupted samples of blended juices.

NMR analyses have also been useful for the identification of several wine products. Recently, the analysis and identification of the products formed during winemaking by using different yeasts within the fermentation process has been assayed. Surprisingly, there were significant differences between the metabolites of the wines produced by the different yeasts [33]. In this regard, ^1^H-NMR spectra evidenced the presence of different concentrations of the evaluated molecules, such as glycerol, 2,3-butanediol, lactic acid, malic acid, tartaric acid, succinic acid, gallic acid, proline, alanine, valine, choline, and ethyl acetate, related to the yeast and used to carry out winemaking. Nevertheless, the compositions of valine, alanine lactic acid, and gallic acid were the most important differences. The latter could be detrimental to the quality of the wine, because the high content of organic acids can cause sour mouthfeels. The method’s sensitivity revealed the importance of the use of different yeast strains for producing specific properties in wines by detecting differences of compounds’ concentrations in the order of hundredths of grams per liter.

NMR spectroscopy has been successful in characterizing key molecules from wine and grapes that positively affect human health. A recent study evaluated Aglianico grape and red wines produced from said raw grapes [34]. They reported several bioactive compounds through a combination of HR-ESI-MS and ^1^H-NMR, such as catechin, epicatechin, gallic acid, 2-phenylethanol, syringic acid, tryptophol, tyrosol, xanthurenic acid, and oleanolic acid. HR-ESI-MS sensitivity combined with unique proton NMR chemical shifts and scalar *J* coupling fingerprints have allowed the identification of the latter two compounds in relatively high concentrations for the first time. Xanthurenic acid has protective effects on key brain functions by interactions with brain receptors and as a regulatory molecule of redox maintenance [35]. Its presence in wines strengthens a moderate consumption of wine as a health-promoting practice. For the case of oleanolic acid (oleanic acid), a correlation between its consumption and improvements in bone properties has been demonstrated, such as an enhancement in the calcium balance shown in rat models [36]. Additionally, its potential therapeutic effects in preventing and managing chronic diseases such as cancer, diabetes, hepatic illnesses, hypertension, and inflammatory processes has been demonstrated [37].

To study nonprofessionalized traditional spirits such as Mexican mezcal, we have recently implemented NMR foodomics to profile the metabolite composition of both ancestral and artisanal products [32]. This work has been possible to discriminate the geographical origin of mezcals from the Oaxaca, Puebla, and San Luis Potosí Mexican regions, from the spectral fingerprint generated by NMR data matrices that reflect the presence of eleven discriminant metabolites in different concentrations. The proton NMR exhibited the presence of acetaldehyde (δ = 9.55 ppm, *J* = 2.82 Hz, quartet), 5-substituted furanaldehyde (δ = 9.37 ppm, singlet; δ = 9.19 ppm, singlet), 2-furfural (δ = 7.97 ppm, multiplet; δ = 7.34 ppm, multiplet), unsubstituted furfural, 2-furoic acid (δ = 6.54 ppm, *J* = 3.6 Hz/1.57 Hz, doublet of doublets), (furan-2-yl)-methanol (δ = 6.24 ppm, *J* = 3.3 doublet), phenethyl alcohol (δ = 7.17 ppm, *J* = 7.47 Hz multiplet), phenethyl acetate (δ = 7.09 ppm, *J* = 7.48 Hz multiplet), ethyl acetate (δ = 3.99 ppm, *J* = 7.2 Hz, quartet), 1-butanol (δ = 1.54 ppm, *J* = 6.67 multiplet), 2-butanol (δ = 1.49 ppm, *J* = 6.67 multiplet), and 2-methylpropan-1-ol (δ = 1.38 ppm, *J* = 7.1 Hz, multiplet). By the identification of these metabolites at different compositions in the mezcal samples analyzed, it is possible to assign a spectral trace because of the species of agave used for the beverage elaboration—that is to say, the use of *Agave potatorum*, *A. angustifolia*, *A. cupreata*, or *A. salmiana* ssp. *crassispina*.

Several reports have emerged over the last years in terms of both NMR acquisitions and MSA combined methodologies for developing different foodomics approaches. The first report applied water-to-ethanol NMR multi-presaturation schemes during mixing times and recovery delays within 1D-NOESY experiments in a set of approximately 600 German wine samples. This data matrix allowed classifying grape varieties, geographical origins, and aging of five wine-growing areas of Southern Germany (Rheinpfalz, Rheinhessen, Mosel, Baden, and Württemberg), with a principal component analysis (PCA), linear discrimination analysis (LDA), and multivariate analysis of variance (MANOVA) [38]. An independent component Analysis (ICA) combined with LDA achieved noticeable improvements to generate discriminative features within the NMR data matrix of German wine samples [39]. To discriminate between Italian “Fiano di Avellino” wines produced with the same grape variety but fermented with commercial or autochthonous yeast starters, the authors used a T_1_-relaxation filter as a strategy for ethanol suppression, instead of water-to-ethanol multi-suppression, proton NMR profiling in combination with PCA, LDA, and a hierarchical cluster analysis (HCA) [40]. Recently, ^1^H-NMR targeted metabolomics has discriminated between Chinese wine regions [41] and grape varieties such as Cabernet Sauvignon, Merlot, and Cabernet Gernischt dry red wines [42], as well as different Chardonnay dry white wines treated with different inactive yeasts before aging [34]. Discriminative features came, respectively, from ethyl acetate, lactic acid, alanine, succinic acid, proline, malic acid, and gallic acid (red wines) and 2,3-butanediol, ethyl acetate, malic acid, valine, succinic acid, lactic acid, tartaric acid, glycerol, gallic acid, choline, proline, and alanine (white wines) spin systems. Furthermore, specific oenological improvements, such as the use of *Hanseniaspora vineae* yeast strains with respect to standard fermentation to enhance aromatic profiles in Spanish Albillo white wines, were evaluated with both ^1^H-NMR and GC-FID-targeted metabolomics [43].

Considering the above-mentioned NMR-MSA state-of-the-art foodomics advances, the present work introduces a set of NMR-MSA novelties. First, the use of a double pulsed-field-gradient echo (DPFGE) experiment [44], with a refocusing band-selective uniform response pure-phase selective pulse [45], for selective excitation of the 5–10-ppm chemical shift range of wine samples, revealed novel broad ^1^H resonances. Second, an NMR-MSA foodomics approach to discriminate between wine samples produced from the same Cabernet Sauvignon variety but fermented with different yeast strains was proposed for large-scale alcohol reductions [46]. The NMR data matrix obtained from DPFGE schemes produces accurate discriminant features for disentangling between standard and (co)inoculation fermentations that afford approximately 1% of alcohol reduction with a diverse Partial Least Squares-Discriminant Analysis (PLS-DA) of DPFGE NMR outliers. Third, different MSA methods afforded a comparative study, such as nonsupervised Principal Component Analysis (PCA), the supervised standard partial (PLS-DA), sparse (sPLS-DA) least squares-discriminant analysis, and orthogonal projections to latent structures discriminant analysis (OPLS-DA). The results have been useful for obtaining holistic fingerprints to discriminate between different Cabernet Sauvignon fermentation schemes and juice varieties (apple, apricot, and orange) or juice products authentications (puree, nectar, concentrated, and commercial juice fruit drinks). NMR data matrices were, respectively, obtained with DPFGE and automatized 1D single-pulse NOESY presaturation NMR schemes.

## 2. Results

### 2.1. Wines’ NMR Outliers with Double Pulsed-Field-Gradient Echo

**Figure 1:**
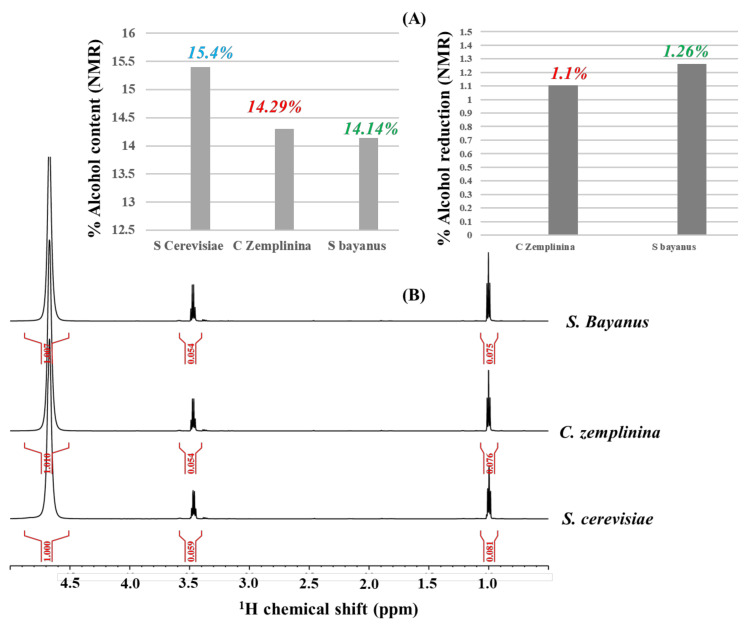

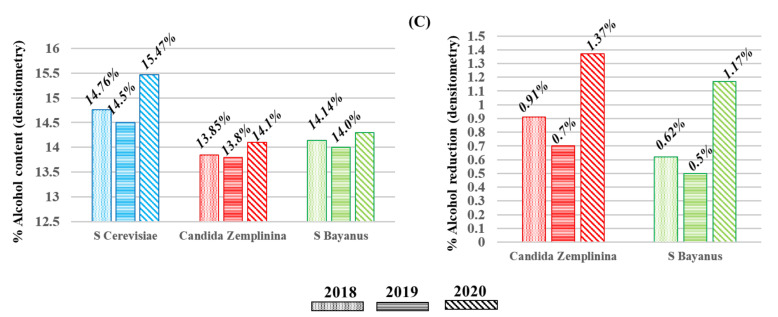
(**A**) The %Alcohol content of Cabernet Sauvignon wine samples, all fermented at a large-scale regime (1950 L produced from c.a. 3000-Kg raw grapes) from the latest 2020 harvest with a standard *Saccharomyces cerevisiae*, co-inoculation with *Candida zemplinina*, and inoculation with *Saccharomyces Bayanus ex uvarum* yeast strains (left). The alcohol reduction with respect to *Saccharomyces cerevisiae* (right), measured with quantitative proton NMR spectroscopy (**B**), in comparison with the quantified %alcohol content (**C**, left) and %alcohol reduction (**C**, right) of the same large-scale fermentation wine samples produced from 2018 (dotted histograms), 2019 (horizontally lined histograms), and 2020 (45° tilted-lined histograms) harvests, all obtained with the standard densitometry technique.

A habitual prerequisite for successfully implementing ^1^H-NMR selective excitation schemes comprises a previous acquisition of a broad-band standard ^1^H direct polarization experiment that can also be afforded in a quantitative way (q-^1^H NMR) [47]. In such conditions that imply previous calibrations of optimal recovery delays and effective hard π/2 pulses, q-^1^H NMR spectroscopy can be used to quantify the %alcohol content in hydroalcoholic solutions [46]. Figure 1B presents q-^1^H NMR spectra of Cabernet Sauvignon wine samples fermented with *Saccharomyces cerevisiae*, *Candida zemplinina*, and *Saccharomyces Bayanus ex uvarum* yeast strains (see Materials and Methods) used as the starting point for optimizing a Double Pulsed-Field-Gradient Echo ^1^H NMR scheme (vide infra), as well as for quantifying their alcohol content (Figure 1A, left), and the %alcohol reduction obtained with *Candida zemplinina* and *Saccharomyces Bayanus ex uvarum* large-scale fermentation schemes (Figure 1A, right). The orthogonality of the %alcohol content and %alcohol reduction measurements with q-^1^H NMR spectroscopy (2020 harvest) was compared with conventional densitometry methods, whereas the alcohol content of the full Cabernet Sauvignon wine sets (Figure 1C, left), as well as alcohol reductions, reached with *Candida zemplinina* and *Saccharomyces Bayanus ex uvarum* (Figure 1C, right) fermentations from the 2018–2020 harvests found accurate agreements with the q-^1^H NMR data.

**Figure 2:**
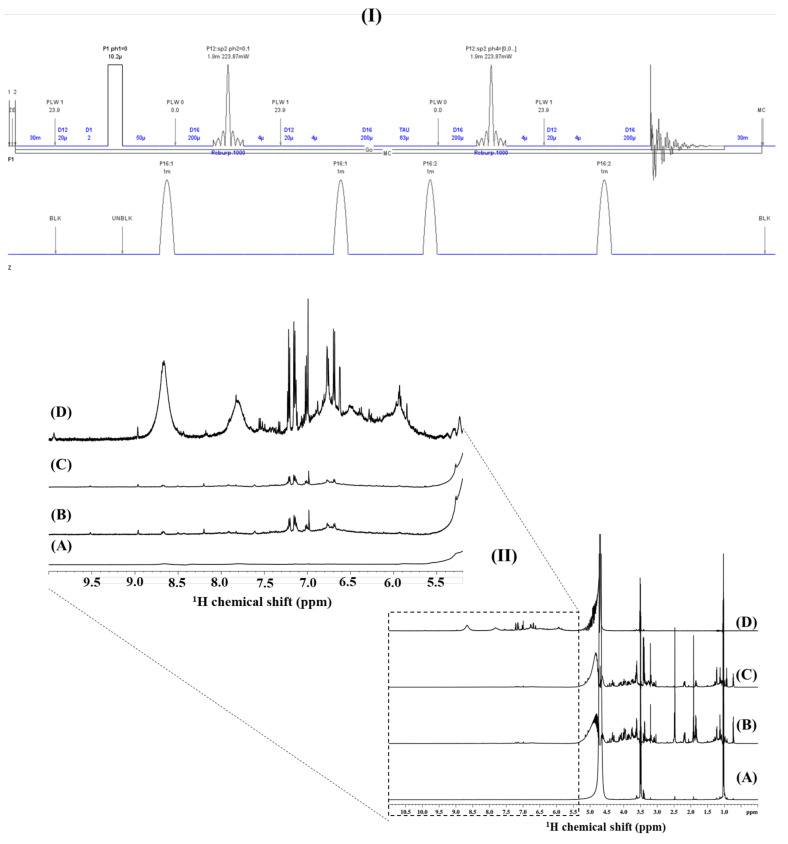
Sensitivity enhancement was obtained in wine samples with a double pulsed-field-gradient echo (DPFGE) proton NMR experiment ((I) and spectrum (**II, D**) with respect to a standard q-^1^H- NMR direct polarization experiment (**II, A**). A {^1^H_water_presat_ NMR}-1D single-pulse NOESY spectrum with an off-resonance shaped-pulse for water and ethanol multi-presaturation during both the relaxation delay and mixing times (**II, B**) [33] and a {^1^H_water_presat_ NMR} spectrum with identical conditions as in (**II, B**) but with an additional continuous-wave decoupling module to eliminate the intense ^13^C satellites of the ethanol signals (**II, C**).

**Figure 3:**
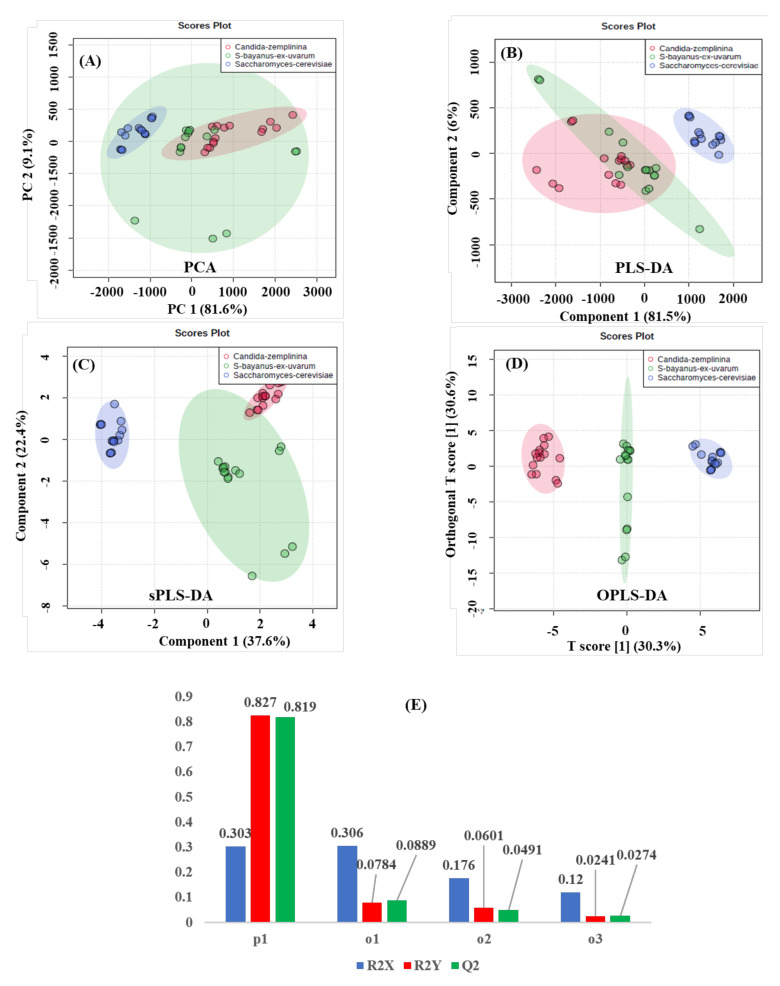
Unsupervised principal component (**A**) and supervised standard partial (PLS-DA) (**B**), sparse (sPLS-DA) (**C**) least square, and orthogonal projections to latent structures discriminant analysis (OPLS-DA) (**D**) score plots of Cabernet Sauvignon wines fermented with *Saccharomyces cerevisiae* (blue)**,** *Candida zemplinina* (red), and *Saccharomyces bayanus ex uvarum* (green) yeast strains, modeled from the DPFGE NMR data matrix (Figure S1). T2 Hotelling’s ellipses have a 95% confidence level in all cases. For PCA, PLS-, and sPLS-DA holistic fingerprints, the explained variances are highlighted in parentheses along the axis. (**E**) For OPLS-DA, the permutation analysis between one predictive (p1) and three orthogonal (o1, o2, and o3) components produced the observed and cross-validated R2X, R2Y, and Q2 coefficients.

Figure 2 shows the clear advantages of applying a Double Pulsed-Field-Gradient Echo (DPFGE) pulse sequence in wine samples. Instead of eliminating the intense signals from the hydroalcoholic solution (i.e., H_2_O singlet at 4.7 ppm with a bandwidth at half-height of c.a. 20 Hz, CH_2_ ethanol quartet at 3.47 ppm, and CH_3_ ethanol triplet at 1.005 ppm; see Figure 1B), a selective π refocusing band-selective uniform response pure-phase (REBURP) pulse [45], flanked by two gradient pulses during an echo period [44], allowed to exclusively refocus the selected chemical shift range comprising the wine aromatic ^1^H spin systems (5.5–11 ppm, 3360 Hz), whilst the rest of the spectrum—including the intense water-to-ethanol hydroalcoholic resonances—are efficiently defocused. The DPFGE NMR scheme (pulse sequence shown within Figure 2, I) used for producing selectively irradiated wine ^1^H NMR spectra (Figure 2, index II, D) is compared with the standard q-^1^H NMR (Figure 2, index II, A) and with two different versions of the {^1^H_water_presat_ NMR }-1D single-pulse NOESY experiment, with an off-resonance shaped-pulse for water and ethanol signal suppression during both the relaxation delay and mixing times (Figure 2, indexes II B and C) [33]. The acquisitions were at equivalent conditions in terms of the number of scans, acquisition times, recovery delays between scans, spectral width, and carrier frequencies to define the offset (see Materials and Methods). The obtained gain in sensitivity with DPFGE spectroscopy was evaluated for the first time as an NMR data matrix (Figure S1) in different unsupervised and supervised multivariate statistical analysis approaches. The MSA approaches afforded the capacity to produce holistic fingerprints that can unambiguously recognize discriminant factors of wines from the same variety but fermented at different conditions (Figure 3)

### 2.2. Juice NMR Outliers with Automatized {^1^H_water_presat_ NMR} -1D Single-Pulse NOESY

Automatized foodomics workflows such as commercial NMR foodscreeners possess considerable advantages from sample to NMR data matrix preparations to carry out multivariate statistical analysis in a precise way, avoiding human inadequacies from multisampling manipulations to spectra acquisition and preprocessing, mostly for nonexpert users. Some possible disadvantages of automatized workflows might be the lack of a wide plethora of methods for obtaining NMR outliers, like, for instance, acquisitions with novel and robust pulse sequences such as DPFGE and spectral preprocessing such as selecting between least squares or parametric time warping NMR alignments and/or free choice for using the most adequate resonances’ bucketing strategy. Another disadvantage of automatized foodomics processes relies on their reduced options for developing multivariate statistical analysis, mostly for third-party alien users out of the context of a foodscreener business model.

**Figure 4:**
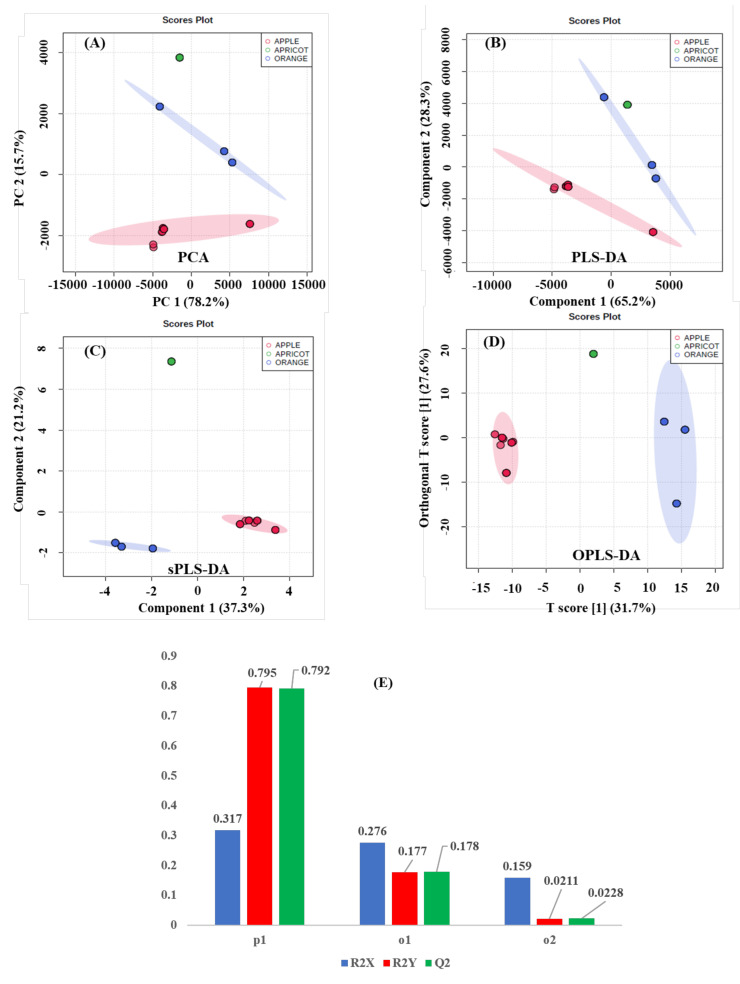
The figure presents the score plots of the unsupervised principal component (**A**) and supervised standard partial (PLS-DA) (**B**), sparse (sPLS-DA) (**C**) least square, and orthogonal projections to latent structures discriminant analysis (OPLS-DA) (**D**). It shows 100% apple juice (red), orange (blue), and apricot (green) reconstituted concentrates (100% juices in different presentations: (a) made from concentrate, (b) commercial, not from concentrate, (c) nectars, and (d) purees, modeled from the automatized {^1^H_water_presat_ NMR} data matrix (Figure S2) as a “type of fruit” holistic fingerprint. T2 Hotelling’s ellipses have a 95% confidence level in all cases. For PCA, PLS-, and sPLS-DA holistic fingerprints, the explained variances are highlighted in parentheses along the axis. (**E**) For OPLS-DA, the permutation analysis between one predictive (p1) and two orthogonal (o1, o2) components produced the observed and cross-validated R2X, R2Y, and Q2 coefficients.

Figure S2 shows series of {^1^H_water_presat_ NMR} spectra of 100% apple, orange, and apricot juices, nectars, and purees (see Material and Methods), obtained from a foodscreener’s automatized and standardized NMR procedure [48]. Targeted NMR analysis of said automatized food screenings, allows to quantify 39 metabolites and physicochemical parameters of commercial fruit juices, (Table 1), typically used as compliance parameters defined at the AIJN Code of Practices of the European Fruit Juice Association, whereas said targeted metabolomics approach is obtained with the use of private consortium databanks [49]. To the best of our knowledge, most of the MSA approaches for obtaining discriminant features from NMR data matrices that are acquired and processed with Standard Operation Procedures in automatized foodscreeners, uses the unsupervised Principal Component Analysis approach. For that, the produced juice {^1^H_water_presat_ NMR} data matrix (Figure 4) related to “type of fruit” and “type of juice” discriminant factors is submitted to different unsupervised (PCA) and supervised (PLS-, sPLS-, and OPLS-DA) multivariate statistical analyses for evaluating the advantages of extending discriminant capacity of automatized NMR outliers, with more robust supervised approaches towards more reliable holistic fingerprints.

### 2.3. Multivariate Statistical Analysis

Both wine DPFGE and juice automatized {^1^H_water_presat_ NMR} data matrices produce specific NMR bins that, respectively, generate 75 and 435 processed data features after data filtering of non-relevant variables (Materials and Methods). Wine and juice postprocessed and normalized NMR outliers are submitted to several multivariate analysis methods. First, the Principal Component Analysis (PCA) explains in an unsupervised way the variance of each dataset when increasing the number of principal components without referring to any class label. The Partial Least Squares–Discriminant Analysis (PLS-DA) extracts the information that can predict all possible class memberships from linear combinations of original NMR bins with the use of multivariate regression techniques, whereas class discriminations are assessed by a permutation test between the original data and the permuted class labels via cross-validations [50]. The Sparse Partial Least Squares–Discriminant Analysis (sPLS-DA) is a special case of PLS-DA for data selection and classification in a one-step procedure, whereas the algorithm is used to effectively reduce an important number of NMR bins, within the original high-dimensional data, for producing robust and easy-to-interpret discriminant models [51]. The Orthogonal Projections to Latent Structures-Discriminant Analysis (OPLS-DA) permits obtaining optimal information from the dataset by identifying a more refined multivariate subspace for maximum group separations by applying Monte-Carlo Cross Validations with a set of partitions per number of permutations [32]. Due to the capacities for distinguishing between subtle variations in NMR datasets that are relevant for keen identifications of spectral features to drive group separations further, OPLS-DA tends to produce less complex discriminant models, with more accurate dimension reductions and more reliable than PLS-DA models [52].

Present work shows strengths and limitations of each above-mentioned MSA approach, by evaluating their discriminant capacity of two different sets of NMR outliers. On the one hand, the DPFGE matrix (Figure S1) discriminates between Cabernet Sauvignon wines fermented at different conditions (*Saccharomyces cerevisiae* co-inoculation with *Candida zemplinina* and inoculation with *Saccharomyces bayanus ex uvarum*) as part of an optimization procedure comprising novel industrial-scale wine alcohol reduction schemes. On the other hand, the automatized {^1^H_water_presat_ NMR} matrix (Figure S2) discriminates “type of fruit” and “type of juice”, i.e., 100% apple, orange, and apricot fruit beverages, in different presentations: (a) made from concentrate, (b) commercial, not from concentrate, (c) nectars, and (d) purees. A targeted analysis of the wine DPFGE matrix will be elsewhere described, as it is currently under development as multidimensional DFPGE NMR schemes for signal assignments. In contrast, Table 1 shows the targeted analysis of commercial apple juices, apple juices made from concentrate, orange juices made from concentrate, and apricot juices made from concentrate using a Bruker Juice Profiling^TM^ remote analysis [53].

## 3. Discussion

### 3.1. Discriminant Analysis of Non-Saccharomyces Large-Scale Alcohol Reductions with Double Pulsed-Field-Gradient Echo NMR Metabolomics

Modern oenological practices have focused on searching strategies for reducing alcohol content in wines, as climate change has induced a significant increase of sugar amount in musts. With conventional anaerobic conditions carried out with *Saccharomyces* yeast strains, fermentation of grapes that have over amounts of sugar content will produce wines with an increased %alcohol content (Figure 1) that, in turn, are translated into products with penalized mouthfeel, taints, and/or flavors, as well as a reduced market consumption and severe tax policies [54]. Among the broad spectrum of viticultural, physical, and microbiological processes recently reported for alcohol reductions, the concatenated use of non-Saccharomyces yeast strains to, first, aerobically sequester the excess of sugar content by respiration by the use of anaerobic *Saccharomyces* strains for final fermentation has gained importance [55,56]. The methods comprising the use of several non-*Saccharomyces* yeast strains for assimilating musts’ sugars to produce wines with reduced alcohol content and appropriate sensorial feelings have been extensively reported at lab, medium, and pilot scales [57,58,59] but scarcely developed at the industrial scale [60].

Herein, we show a set of novel NMR applications to evaluate different fermentation schemes related to alcohol reductions in a large-scale regime. First, q-^1^H NMR [46] evaluates alcohol reductions reached with a large-scale co-inoculation with *Candida zemplinina*, with further anaerobic fermentation with *Saccharomyces cerevisiae*, as well as a large-scale fermentation with a mixed strain containing *Saccharomyces uvarum* and *Saccharomyces cerevisiae ex ph. r. bayanus* (from now on called *Saccharomyces Bayanus ex uvarum* yeast; see Materials and Methods), with respect to a standard fermentation carried out with *Saccharomyces cerevisiae* at the same large-scale conditions. Expected alcohol reductions within a range of 1% were obtained for wines fermented with *Candida zemplinina* and *Saccharomyces Bayanus ex uvarum* from Cabernet Sauvignon grapes obtained from a 2020 harvest. %Alcohol content and %alcohol reduction measured with q-^1^H NMR (Figure 1A) were in agreement with the data measured with the standard densitometry method (45° tilted-lined histograms; Figure 1C) that was, in turn, used to measure the large-scale alcohol reduction efficiency from 2018 (dotted histograms; Figure 1C) and 2019 (horizontally lined histograms; Figure 1C) essays.

q-^1^H NMR serves, in turn, as the starting point to implement the Double Pulsed-Field-Gradient Echo experiment (Figure 2, top). Taking the same spectral width (13 ppm), transmitter frequency offset (4.5 ppm), and π/2 nutation frequency optimized values for broadband hard pulses (10.55 μs @ 24 watts; see Figure S3) as for q-^1^H NMR allows implementing the optimized conditions of the REBURP selective π refocusing band-selective uniform response pure-phase pulse. Selective excitation of the chemical shift range comprising wine aromatic ^1^H spin systems (5.5–11 ppm) with the REBURP-refocusing pulse during the echo period includes optimal REBURP pulse lengths (1900 ms), power levels (0.223 watts), and irradiation bandwidths (3360 Hz) values. The latter is defined in terms of a frequency offset of the shaped pulse, defined at +2300 Hz with respect to the carrier frequency at 4.5 ppm (i.e., at 8.35 ppm (see Materials and Methods and the REBURP excitation profile obtained with NMR simulations; Figure S4). ^1^H-DPFGE spectra (Figure 2D) notably increases the signal-to-noise ratio of aromatic spin systems (S/N = 68.72 obtained with an optimized receiver gain of 203; see also Figure S5) with respect to the standard q-^1^H direct polarization NMR spectrum (S/N = 33.59 obtained with an optimized receiver gain of 1; Figure 2A and Figure S5), a {^1^H_water_presat_ NMR}-1D single-pulse NOESY spectrum with an off-resonance shaped-pulse for water and ethanol multi-presaturation during both the relaxation delay and mixing times (S/N = 10.3 obtained with an optimized receiver gain of 40.3; Figure 2B and Figure S5), and a {^1^H_water_presat_ NMR} spectrum with identical conditions as in the former case but with an additional continuous-wave decoupling module to eliminate intense ^13^C satellites of ethanol signals (S/N = 15.23 obtained with an optimized receiver gain of 25.4; Figure 2C and Figure S5). A clear advantage of the DPFGE experiment in oenological NMR spectroscopy is that the selective refocusing of the aromatic chemical shift range (5.5–11 ppm, 3360 Hz) with an efficient defocusing of the water-to-ethanol hydroalcoholic resonances permits the use of maximal receiver gain values that produces a signal-to-noise ratio that reveals novel broad ^1^H aromatic resonances strongly related to complex polyphenols. However, their targeted analysis will be elsewhere published by reporting the advantages of implementing DPFGE multidimensional NMR spectroscopy. Currently, the discriminant capacity of the novel DPFGE NMR data matrix (Figure S1) was presented to discriminate between Cabernet Sauvignon wines fermented at specific large-scale conditions (See Results and Materials and Methods), whereas the data dimensionality of said DPFGE NMR outliers was obtained with an Intelligent Binning Method [61], in turn stressed with gray boxes in Figure S1.

Both unsupervised (Figure 3A) and supervised (Figure 3B–D) multivariate statistical nontargeted analyses of the binned DPFGE NMR data matrix are schemed in Figure 3. An unsupervised principal component analysis is generally used to organize the NMR data matrix and determine correlations between selected factors (large-scale fermentation scheme) and outliers (discriminant DPFGE NMR resonances). First, the most significative Principal Components (PC1 = 81.6% and PC2 = 9.1%) with a 90.7% variance of the unsupervised PCA (Figure 3A) does not allow significant discriminations between Cabernet Sauvignon wines fermented with *Saccharomyces cerevisiae* (blue T2 Hotelling’s ellipses with a 95% confidence level), *Candida zemplinina* (red T2 Hotelling’s ellipses with a 95% confidence level), and *Saccharomyces bayanus ex uvarum* (green T2 Hotelling’s ellipses with a 95% confidence level) yeast strains. Consequently, supervised MSA methods were applied, looking forward to increasing the discriminant capacity of the DPFGE data matrix. In contrast to the PCA, supervised PLS-DA (Figure 3B) provides discrimination between standard *Saccharomyces cerevisiae* (blue T2 Hotelling’s ellipses with a 95% confidence level) and non-*Saccharomyces* yeast strains used for large-scale alcohol reductions (*Candida zemplinina*, with red T2 Hotelling’s ellipses defining a 95% confidence level and *Saccharomyces bayanus ex uvarum*, with green T2 Hotelling’s ellipses defining a 95% confidence level), with the most significative score plots (PC1 = 81.5% and PC2 = 6.0%) and an overall 87.5% variance. Interestingly, the DPFGE NMR data matrix’s discriminant capacity between conventional and alcohol reduction fermentation schemes is also represented with the sPLS-Discriminant Analysis (Figure 3C), whereas, with a sparse number of variables of the DPFGE data dimensionality that produce a 60% overall variance by considering the most representative PC1 (37.6%) and PC2 (22.4%) score plot components, discriminations between Cabernet Sauvignon fermentations with standard *Saccharomyces cerevisiae* and non-*Saccharomyces* yeast strains for alcohol reductions are affordable. Finally, OPLS-DA permits obtaining optimal information from the dataset by identifying a more refined multivariate subspace for maximum group separations by applying Monte-Carlo cross-validations with a set of partitions per number of permutations. OPLS-DA modeling was applied to obtain improved separations amongst used yeast strains that allowed pairwise comparisons of discriminative features between fermentation processes. Discriminations between Cabernet Sauvignon wines fermented with *Saccharomyces cerevisiae* (blue T2 Hotelling’s ellipses with a 95% confidence level), *Candida zemplinina* (red T2 Hotelling’s ellipses with a 95% confidence level), and *Saccharomyces bayanus ex uvarum* (green T2 Hotelling’s ellipses with a 95% confidence level) yeast strains by a supervised OPLS-DA discriminative analysis are highlighted in Figure 3D, whereas the permutation test with one predictive and three orthogonal components revealed the high statistical discriminant capacity of the DPFGE NMR outlier when analyzed with a supervised orthogonal projection to the latent structures discriminant model: R2X: 0.905, R2Y: 0.9896, and Q2: 0.9844 (Figure 3E).

A particular limitation of the presented DPFGE acquisition approach must be taken into consideration. Selective excitation of any desired chemical shift range with the REBURP refocusing pulse during an echo period is effective over an irradiation bandwidth with respect to a selected frequency offset of the shaped pulse, as above stressed. Said frequency offset of the REBURP selective pulse is, in turn, defined with respect to the transmitter frequency offset (4.5 ppm in the present work), whereas, in several reports, is nevertheless defined at the center of the isotropic chemical shift of the water signal (4.7 ppm at standard temperature and buffered conditions). Special care must be taken when known instrumental distortions occur, such as radiation damping effects [62], that typically shifts water resonances out of the expected chemical shift value, which can severely affect the efficiency of the REBURP selective irradiation if the frequency offset of the shaped pulse is defined with respect to the center of the water signal. As known, radiation damping effects are stronger at 600 MHz or higher magnetic fields and the use of cryoprobes. In summary, the REBURP selective excitation efficiency during a DPFGE experiment might be severely hampered by offset inhomogeneities.

### 3.2. Discriminant Analysis between “Type of Fruit” and “Type of Juice” in Different 100% Apple, Orange, and Apricot Beverages with Automatized {^1^H_water_presat_ NMR} Metabolomics

As with the DPFGE NMR data matrix (Figure S1), the data dimensionality of automatized {^1^H_water_presat_ NMR} juice outliers are obtained with an Intelligent Binning Method [61], which was applied to the full chemical shift range of juice NMR spectra (see gray buckets for the aromatic moiety in Figure S2).

**Figure 5:**
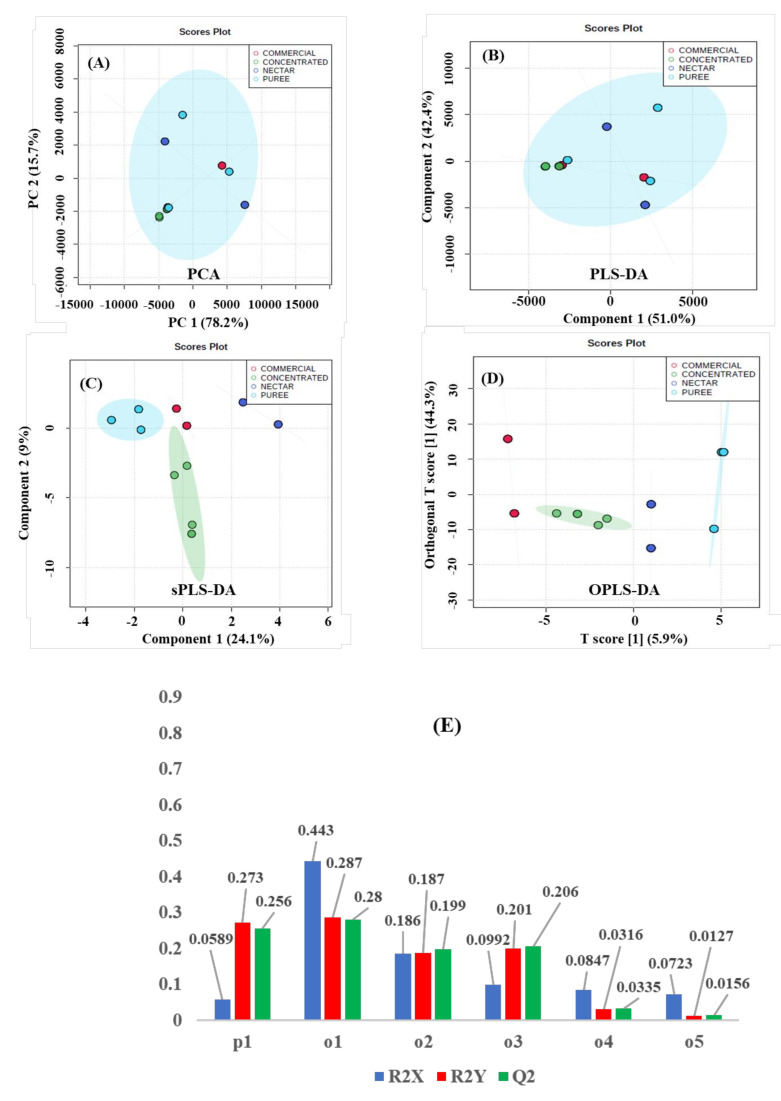
Score plots of the unsupervised principal component (**A**) and supervised standard partial (PLS-DA) (**B**), sparse (sPLS-DA) (**C**) least square, and orthogonal projections to latent structures discriminant analysis (OPLS-DA) (**D**). It shows commercial nonconcentrated juice (red); concentrated juice (green); nectar (blue); and puree (cyan) fruit drinks (apple, apricot, and orange), modeled from the automatized {^1^H_water_presat_ NMR} data matrix (Figure S2) as the “type of juice” holistic fingerprint. T2 Hotelling’s ellipses have a 95% confidence level in all cases. For PCA, PLS-, and sPLS-DA holistic fingerprints, the explained variances are highlighted in parentheses along the axis. (**E**) For OPLS-DA, a permutation analysis between one predictive (p1) and five orthogonal (o1, o2, o3, o4, and o5) components produced the observed and cross-validated R2X, R2Y, and Q2 coefficients.

Unsupervised (Figure 4A and Figure 5A) and supervised (Figure 4B–D and Figure 5B–D) multivariate statistical nontargeted analyses of the binned {^1^H_water_presat_ NMR} data matrix were applied for determining correlations between selected factors (type of fruit and type of juice in apple, apricot, and orange beverages) and outliers (discriminant {^1^H_water_presat_ NMR} shifts). The unsupervised PCA (Figure 4A) for fruit beverages gives the most significant Principal Components (PC1 = 78.2% and PC2 = 15.7%) with a 93.79% variance. It enables to make significant discriminations between orange (blue T2 Hotelling’s ellipses with a 95% confidence level), apple (red T2 Hotelling’s ellipses with a 95% confidence level), and apricot (green T2 Hotelling’s ellipses with a 95% confidence level) juice beverages. Supervised MSA methods are applied in an attempt to perform the discriminant capacity of {^1^H_water_presat_ NMR} data matrix. Supervised PLS-DA (Figure 4B) provides comparable discriminations to PCA amongst apple, apricot, and orange beverages by analyzing the score plots (PC1 = 65.2% and PC2 = 28.3%) and its overall 93.5% variance. Furthermore, the {^1^H_water_presat_ NMR} data matrix’s discriminant capacity between the type of fruit in juice analyses is also represented with the sPLS-Discriminant Analysis (Figure 4C). Discriminations between apple, apricot, and orange fruit beverages are as well-affordable with a sparse number of variables of the {^1^H_water_presat_ NMR} data dimensionality that produce a 58.5% overall variance by considering the most representative PC1 (37.3%) and PC2 (21.2%) score plot components. Finally, OPLS-DA modeling is applied for obtaining discriminations between the type of fruit within analyzed beverages (Figure 4D). The permutation test with one predictive and two orthogonal components reveals high statistical discriminant capacity of the {^1^H_water_presat_ NMR} outlier when analyzed with a supervised orthogonal projection to latent structures discriminant model: R2X: 0.752, R2Y: 0.9931, and Q2: 0.9928 (Figure 4E).

Figure 5 comprises MSA of commercial nonconcentrated juice (red score plots), concentrated juice (green score plots), nectar (blue score plots), and puree (cyan score plots and T2 Hotelling’s ellipse with a 95% confidence level) fruit drinks. An unsupervised analysis shows that the most significant Principal Components (PC1 = 78.2% and PC2 = 15.7%) do not produce significant discriminations with a 93.79% PCA variance (Figure 5A). The supervised MSA methods are applied for performing the discriminant capacity of {^1^H_water_presat_ NMR} data matrix towards the “type of juice” discriminant factor. Supervised PLS-DA (Figure 5B) provide as well limited discriminations amongst juices in different presentations: (a) made from concentrate, (b) not from concentrate (commercial; see Table 1), (c) nectars, and (d) purees, with the most significant score plots (PC1 = 51% and PC2 = 42.4%) and an overall 93.4% variance. In clear contrast, the {^1^H_water_presat_ NMR} data matrix’s discriminant capacity between type of juice analysis is notably enhanced with the sPLS-Discriminant Analysis (Figure 5C). The sPLS-Discriminant Analysis with a sparse number of variables of the {^1^H_water_presat_ NMR} data dimensionality produced 33.1% overall variance by considering the most representative PC1 (24.1%) and PC2 (9.0%) score plot components, enabling clear discriminations between juices made from concentrate, commercial not from concentrate, nectars, and purees. Finally, OPLS-DA modeling was applied for obtaining discriminations between the type of juice in apple, apricot, and orange beverages (Figure 5D). The permutation test with one predictive and five orthogonal components revealed an acceptable statistical discriminant capacity of the {^1^H_water_presat_ NMR} outlier when analyzed with a supervised orthogonal projection to latent structures discriminant model: R2X: 0.9441, R2Y: 0.9923, and Q2: 0.9901 (Figure 5E).

The juice automatized {^1^H_water_presat_ NMR} data matrix possesses a fair set of discriminant features (*vide infra*) for clearly distinguish between orange, apple, and apricot “type of fruit” analyzed beverages with all herein used MSA approaches, whereas even the unsupervised PCA produces a discriminant holistic fingerprint amongst batches (PC1 = 78.2% and PC2 = 15.7% with a 93.79% variance). Said discriminant capacity of the automatized {^1^H_water_presat_ NMR} data matrix is confirmed in the rest of supervised MSA approaches and, particularly, with OPLS-DA, whereas a minimal permutation test with one predictive and two orthogonal components produces a “type of fruit” discriminant holistic fingerprint between analyzed juice batches, with an acceptability of Q2 = 0.9928. In clear contrast, the same automatized {^1^H_water_presat_ NMR} data matrix is limited to discriminate between “type of juices” with unsupervised PCA and supervised PLS-DA (vide supra). As observed in Figure 5D, OPLS-DA offers an option to discriminate between “type of juices.” However, the OPLS-DA permutation test with the extensive use of five orthogonal components per permutation for providing acceptability of Q2 = 0.9901 indicates the forced way to provide a discriminant model amongst “type of juices” with the use of said important number of orthogonal components. Finally, the striking differences between overall variances obtained with sPLS-DA models for both juice discriminant factors, respectively, 58.5% (“type of fruit”) and 33.1% (“type of juices”), clearly demonstrate the accuracy of the {^1^H_water_presat_ NMR} data matrix to discriminate between fruits and the limitations to discriminate between type of juices.

**Figure 6:**
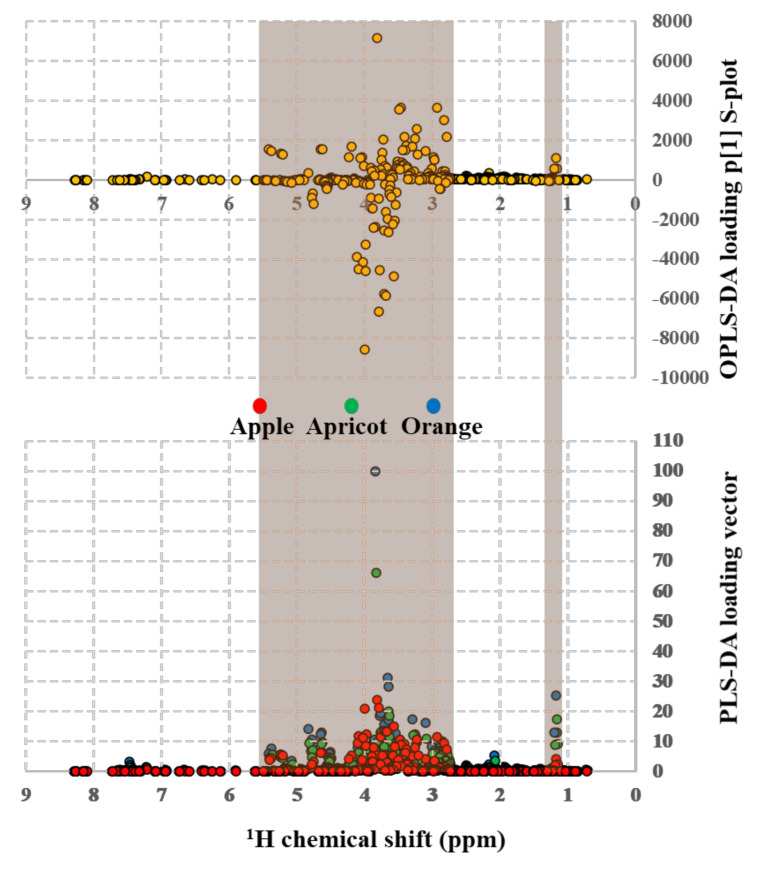
OPLS-DA loading p [1] S-plot (**top**) and PLS-DA loading vectors (**bottom**) as a function of {^1^H_water_presat_ NMR} chemical shifts from 100% apple (red), orange (blue), and apricot (green) the fruit beverage supervised discriminant analysis (Figure 4B,D) that indicates the importance of relevant metabolites used for “types of fruit” in juice discriminations.

**Figure 7:**
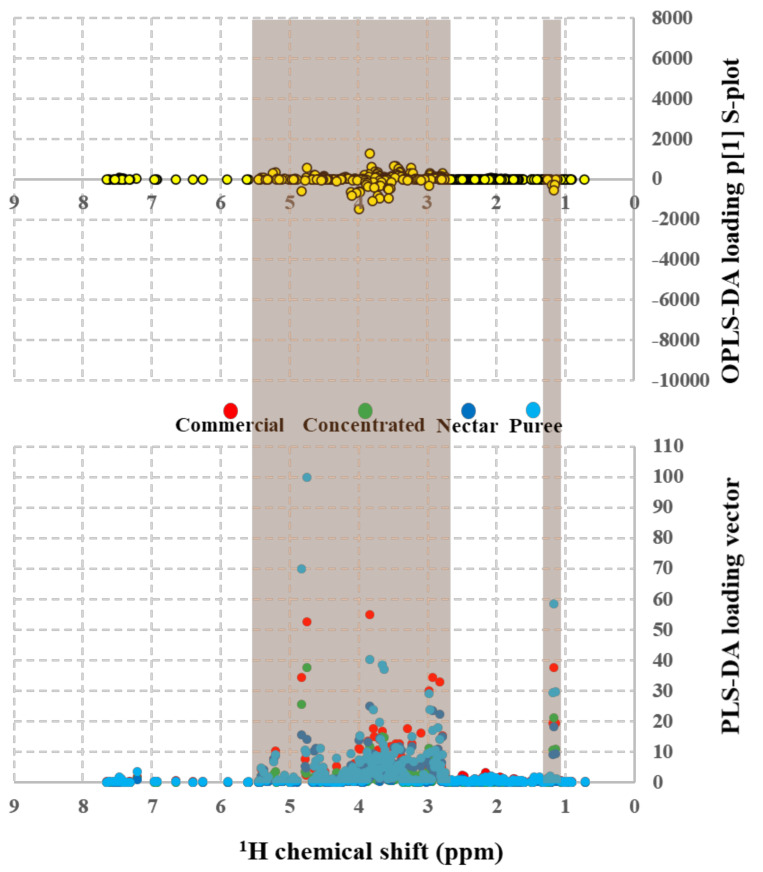
OPLS-DA loading p [1] S-plot (**top**) and PLS-DA loading vectors (**bottom**) as a function of {^1^H_water_presat_ NMR} chemical shifts from the commercial nonconcentrated juice (red); concentrated juice (green); nectar (blue); and puree (cyan) fruit drinks (apple, apricot, and orange) supervised discriminant analysis (Figure 5B,D) that indicates the importance of relevant metabolites used for “type of juice” discriminations.

The importance of each binned chemical shift from {^1^H_water_presat_ NMR} data matrix for carrying out “type of fruit” and “type of juice” discriminative analysis with supervised MSA, is evidenced with PLS-DA loading vectors and OPLS-DA loading p [1] S-plots as a function of ^1^H chemical shifts (Figure 6 and Figure 7). In both pairs of loading plots, discriminant ^1^H shifts (in turn related to specific fruit beverage metabolites) that define the variable importance in “type of fruit” (Figure 6) and “type of juice (Figure 7) discriminant models belong to a chemical shift range between 2.63 to 5.59 ppm and 1.02–1.3 ppm (orange regions in Figure 6 and Figure 7). In agreement with previous reports, said chemical shift regions contain resonances of juice relevant metabolites, such as anomeric glucose (5.17 and 4.7 ppm) spin systems, as well from sucrose (c.a. 5.3 ppm and 4.2 ppm), fructose (4.1 ppm), malic acid (4.45 and 2.75 to 2.85 ppm), succinic acid (2.65 ppm), and ethanol (1.1 ppm) proton shifts [23,53,63]. Table 1 shows the relevant identified metabolites and their quantification. In the case of apple juice, a targeted analysis (Table 1) shows most values at acceptable ranges [49], except the magnesium content, which is slightly low compared with the AIJN value. Moreover, the apple concentrated beverage presents values for malic acid, glucose, fructose, and alanine out of AIJN range by more than 10% due to defect. The orange concentrates show six parameters out of AIJN range, with a downward deviation minor or equal to 10% comprising glucose and 4-aminobutanoic acid, and with an upward deviation minor or equal to 10% are sucrose, alanine, proline, and the sucrose percentages that have greater deviations. Finally, in the apricot concentrate, malic acid shows a slightly low value, but alanine concentration has a deviation greater than 10% of the lower limit of the AIJN range.

## 4. Materials and Methods

### 4.1. Materials

#### 4.1.1. Cabernet Sauvignon Wines Fermented at Different Large-Scale Conditions

Cabernet Sauvignon grape varieties (*Vitis vinifera L.*) were fermented at the “Casa Madero” winery in Parras, Coahuila, Mexico at a large-scale regime for obtaining approximately 1950 L of wine, using c.a. 3000 kg of raw grapes, in three consecutive years of vintage (2018, 2019, and 2020). The yeast strain *Saccharomyces cerevisiae* used as the control comprised the branded D254^TM^ industrial wine yeast (Lallemand, Montreal, QC, Canada).

Large-scale alcohol reductions were carried out with two different procedures:

A first-step co-inoculation with Non-*Saccharomyces Candida zemplinina* (Enantis Ferm) yeast strain, followed by a later inoculation with a D254^TM^ industrial wine yeast *Saccharomyces cerevisiae* (Lallemand, Montreal, QC, Canada).

Inoculation with a mixed strain containing *Saccharomyces uvarum* and *Saccharomyces cerevisiae ex ph. r. bayanus* (herein mentioned as *Saccharomyces Bayanus ex uvarum*) yeast strain (EnartisFerm ES U42).

Microbiological and oenological characteristics of the Non-*Saccharomyces Candida zemplinina* (Enantis Ferm) and hybrid *Saccharomyces uvarum* with *Saccharomyces cerevisiae ex ph. r. bayanus* yeast strains are reported in Tables S1 and S2. Brix degrees of raw grape juices, as well as Cabernet Sauvignon wine pH, total acidity, and free sulfites of each 2018–2020 large-scale fermentation, are reported in Figure S5.

#### 4.1.2. Apple, Orange, and Apricot Juice Rroducts: (a) Made from Concentrate, (b) Not from Concentrate, (c) Nectars, and (d) Purees

All fruit products herein analyzed comprising 100% apple juice not from concentrate (del Valle^TM^), apple nectar (Jumex^TM^), 100% apple puree (Jumex^TM^), 100% apricot puree (Jumex^TM^), 100% orange juice not from concentrate (Jumex^TM^), orange nectar (Jumex^TM^) and 100% orange puree (Jumex^TM^) were commercially obtained.

### 4.2. Methods

#### 4.2.1. NMR Spectroscopy of the Cabernet Sauvignon Wine Samples Fermented with Saccharomyces Cerevisiae, Candida Zemplinina, and Saccharomyces Bayanus Ex Uvarum Yeast Strains

For wine batches, 540 μL of wines were dissolved in 60 μL of deuterium oxide solution, with 99.9% deuteration mixed with 0.05 wt% of 3-(trimethylsilyl) propionic-2,2,3,3-d4 acid and sodium salt as the internal reference (CAS No. 7789-20-0), and 0.1% of phosphonate KH_2_PO_4_ (CAS No. 7778-70-0) buffer was prepared and pH-adjusted to a value of 3.1.

All wine NMR spectra were recorded at 14.1 Teslas of static magnetic field on a Bruker 600 AVANCE III HD equipped with a 5-mm 1H/D BBO probe head with z-gradient. The following set of NMR experiments were conducted:

(a) Standard quantitative ^1^H-one dimensional direct polarization NMR experiments (q-^1^H-NMR; Figure 1B and Figure 2A) were carried out for measuring the %alcohol content and %alcohol reductions reached with *Candida zemplinina* and *Saccharomyces bayanus ex uvarum* yeast strains by previously calibrating the 90° hard pulse (10.55 μs @ 23.69 kHz; see Figure S1) by using 16 transients of 65,536 complex points, having recycling delays of 15 s and with acquisition times of 1723 ms, which produced experimental times of 16 min, 43 s per spectrum. No apodization function was applied during Fourier-Transform.

(b) {^1^H_water_presat_ NMR} (Figure 2B): 1D single-pulse NOESY spectra with an off-resonance shaped-pulse water-to-ethanol multi-presaturation during the relaxation delay (3 s), mixing times (100 milliseconds), and power level irradiation amplitudes of 1.04 × 10^−3^ W for solvent multi-suppression. The acquisitions were as follows: 64 transients were collected into 65,536 complex data points, with acquisition times of 1500 ms producing an experimental time of 4 min and 30 s.

(c) {^1^H_water_presat_ NMR with continuous wave (CW) decoupling on ^13^C channel} (Figure 2C): 1D single-pulse NOESY spectra with an off-resonance shaped-pulse water-to-ethanol multi-presaturation during both the relaxation delay (3 s) and mixing times (100 milliseconds). A CW decoupling module to eliminate intense ^13^C satellites of ethanol signals and with power level irradiation amplitudes of 2.51 × 10^−3^ W for solvent multi-suppression. The acquisitions were as follows: a total of 64 transients were collected into 32,768 complex data points, with acquisition times of 800 ms producing an experimental time of 4 min and 3 s.

(d) Selective Double Pulsed-Field-Gradient Echo (DPFGE) ^1^H NMR spectra (Figure 2D and Figure 3) for the selective excitation of aromatic ^1^H spin systems (5.5–11 ppm, 3360 Hz). The acquisitions were as follows: from an optimized q-^1^H NMR with a spectral width of 13 ppm and a transmitter frequency offset of 4.5 ppm, the pulse sequence schemed in Figure 2, top was implemented. The REBURP selective π refocusing band-selective uniform response pure-phase pulse that is flanked by two gradient pulses during an echo period that allows to exclusively refocus the selected aromatic chemical shift range whilst it is simultaneously defocusing the intense water-to-ethanol hydroalcoholic chemical shift range was calibrated with the aid of the programs Shape tool and NMRSIM (Bruker Biospin; see Figure S2) in order to selectively excite a frequency range of 3360 Hz from a frequency offset of the REBURP pulse, defined at +2300 Hz with respect to the carrier frequency at 4.5 ppm that allowed exciting a chemical shift range between 5.5–11 ppm. The pulse length of the REBURP π pulse was defined at 1900 ms with a power level of 0.223 watts. The acquisitions were as follows: 64 transients were collected into 262,144 complex data points, with acquisition times of 3076 ms and recovery delays of 2 s, and produced experimental times per wine batch of 5 min and 24 s.

#### 4.2.2. NMR Spectroscopy of Apple, Orange, and Apricot 100% Juices Made from Concentrate, Not from Concentrate, Nectars, and Purees

For juice batches, 900 µL of juice were manually mixed with 100 µL of deuterated buffer food type A (H129666) or type B (H129667), depending on the type of fruit. Juice NMR spectroscopy was carried out in a Bruker Avance III Foodscreener spectrometer (Bruker, Karlsruhe, Germany), operating at 400.13 MHz proton frequency, equipped with a SampleXpress 5-mm tube autosampler. Automatized {^1^H_water_presat_ NMR} spectra (Figure S1) comprised a 1D single-pulse NOESY spectra with an off-resonance shaped-pulse water presaturation during both the relaxation delay (4 s) and mixing times (60 milliseconds) and with power level irradiation amplitudes of 3.2 × 10^−5^ W for the solvent suppression. The acquisitions were as follows: a total of 16 transients were collected into 131,072 complex data points, with acquisition times of 3984 ms, produced an experimental time of 2 min and 7 s.

#### 4.2.3. H-NMR Postprocessing and Multivariate Statistical Analysis (MSA)

NMR postprocessing for producing the MSA input variables was carried out as follows: ppm calibration and manual phase corrections were conducted using Bruker TopSpin 4.0.8 software. Global and intermediate baseline corrections, least-squares or parametric time warping NMR alignments, variable size bucketing for untargeted profiling, and data matrix normalization were carried out with NMRProcFlow software [64]. Scaling and statistical analysis workflow for obtaining the Principal Component (PCA Figure 3A, Figure 4A, Figure 5A); standard (PLS-DA; Figures Figure 3B, Figure 4B, Figure 5B); and sparse partial least-square discriminant analysis (sPLS-DA; Figures Figure 3C, Figure 4C, Figure 5C), as well as the Orthogonal Projections to Latent Structures Discriminant Analysis (OPLS-DA; Figure 3D, Figure 4D, Figure 5D) from the constant sum normalized DPFGE (Figure 3) and automatized {^1^H_water_presat_ NMR} (Figure 4) data matrix, was developed with MetaboAnalyst 5.0 software [65]. In all cases, T2 Hotelling’s regions depicted by ellipses in score plots of each model defined a 95% confidence interval [66]. Supervised OPLS-DA was carried out with Monte-Carlo cross-validations with 10 test partitions per 100 permutations for testing [67]. The OPLS-DA, R^2^X, R^2^Y, and Q^2^ statistical parameters that defined the quality of each model were expressed in Figure 3E, Figure 4E, Figure 5E [61].

## 5. Conclusions

The present work describes the use of unsupervised and supervised multivariate statistical analyses applied in two different NMR data outliers. First, the use of a double pulsed field-gradient-echo (DPFGE) NMR methodology, applied for the first time, as a selective refocusing method of the aromatic frequency range comprised between 5.5 and 10 ppm of the wine samples fermented at different conditions, revealed novel discriminant resonances. Supervised standard and sparse PLS-DA multivariate statistical treatments applied in DPFGE matrices produced holistic fingerprints that disentangled between standard and novel large-scale fermentation schemes designed for obtaining approximately 1% of alcohol reductions. The more robust supervised OPLS discriminant analysis of the DPFGE NMR data matrices produced a score plot that clearly discriminated each yeast strain used for the controls (*Saccharomyces cerevisiae*) and large-scale alcohol reductions (*Candida zemplinina* and *Saccharomyces Bayanus ex uvarum*). A supervised multivariate statistical analysis of DPFGE NMR data showed their potential as a milestone in the future optimizations of large-scale alcohol reductions for the selection of equivalent and even better yeast strains that can significantly reduce the alcohol content in wines fermented from grapes affected by climate change. Furthermore, routine unsupervised and extended supervised PLS-DA, sPLS-DA, and OPLS-DA multivariate analyses were as well-applied in automatized {^1^H_water_presat_ NMR} outliers for juice analyses. For the “type of fruit” discriminant analysis, all MSA approaches can easily discriminate between apple, apricot, and orange raw materials. In contrast, only supervised sPLS-DA and OPLS-DA approaches produced significant discriminations between commercial nonconcentrated and concentrated juices, as well as between nectars and purees. Although automated methods possess many advantages, mostly for nonexpert users looking forward to “push one button” solutions, nonautomated/nonstandard methods may have the advantage of using robust NMR pulse sequences or multivariate statistical analyses to obtain more reliable holistic fingerprints than automated methods.

## Figures and Tables

**Table 1:** Apple, orange, and apricot juice targeted analyses, comprising the quantification of 39 metabolites and physicochemical parameters (titratable acidity) used as compliance parameters defined at the AIJN Code of Practices of the European Fruit Juice Association [49].

Compound	Commercial Apple Juice (Not from Concentrate)		Apple Juice Made from Concentrate		Orange Juice Made from Concentrate		Apricot Juice Made from Concentrate	
	Conc.	Unit	Conc.	Unit	Conc.	Unit	Conc.	Unit
Ethanol	195	mg/L	<10	mg/L	<10	mg/L	11	mg/Kg
Lactic acid	66	mg/L	<10	mg/L	77	mg/L	<20	mg/Kg
5-Hydroxymethylfurfural	6	mg/L	<5	mg/L	<5	mg/L	<5	mg/Kg
Citric acid	<0.5	g/L	<0.5	g/L	6.7	g/L	7.2	g/Kg
Malic acid	4.3	g/L	1.6	g/L ↓	1.5	g/L	4.9	g/Kg ↓
Glucose	21.5	g/L	8.6	g/L ↓	18.6	g/L ↓	32.2	g/Kg
Fructose	62.9	g/L	21.8	g/L ↓	20.9	g/L	19.5	g/Kg
Glucose/fructose ratio	0.34		0.39		0.89		1.65	
Sucrose	14.1	g/L	7	g/L	50.1	g/L ↑	16	g/Kg
% Sucrose	14	%	19	%	56	% ↑	24	%
Total sugar	98.6	g/L	37.3	g/L	89.6	g/L	67.7	g/Kg
Alanine	20	mg/L	<5	mg/L ↓	39	mg/L ↓	28	mg/Kg ↓
Acetaldehyde	<5	mg/L	<5	mg/L	<5	mg/L	<5	mg/Kg
Benzoic acid	<10	mg/L	<10	mg/L	<10	mg/L	<10	mg/Kg
Formic acid	<5	mg/L	<5	mg/L	<5	mg/L	5	mg/Kg
Methanol	44	mg/L	<10	mg/L	<10	mg/L	40	mg/Kg
Sorbic acid	<10	mg/L	<10	mg/L	<10	mg/L	<10	mg/Kg
Succinic acid	16	mg/L	<10	mg/L	17	mg/L	32	mg/Kg
Benzaldehyde	<5	mg/L	<5	mg/L	<5	mg/L	ND	
Proline	<50	mg/L	<50	mg/L	366	mg/L ↓	ND	
Galacturonic acid	648	mg/L	<100	mg/L	<150	mg/L	ND	
Acetone	<10	mg/L	<10	mg/L	ND		ND	
Arbutin	<10	mg/L	<10	mg/L	ND		ND	
Chlorogenic acid	28	mg/L	53	mg/L	ND		ND	
Citramalic acid	33	mg/L	<10	mg/L	ND		ND	
Malic/Quinic ratio	17.4	mg/L	20.5	mg/L	ND		ND	
Fumaric acid	<5	mg/L	<5	mg/L	ND		ND	
Pyruvic acid	<10	mg/L	<10	mg/L	ND		ND	
Quinic acid	247	mg/L	76	mg/L	ND		ND	
Xylose	529	mg/L	<300	mg/L	ND		ND	
Potassium *	1016	mg/L	ND		ND		ND	
Magnesium *	39	mg/L ↓	ND		ND		ND	
Titratable Acidity pH 7 *	51	meq/L	ND		ND		ND	
Titratable Acidity pH 8.1 *	52	meq/L	ND		ND		ND	
Titratable Acidity (pH 7, tartaric acid) *	3.8	g/L	ND		ND		ND	
Titratable Acidity (pH 7, malic acid) *	3.3	g/L	ND		ND		ND	
Titratable Acidity (pH 8.1, citric acid) *	3.3	g/L	ND		ND		ND	
4-Aminobutanoic acid	ND		ND		163	mg/L ↓	ND	
Arginine	ND		ND		622	mg/L	ND	
Phlorin	ND		ND		<10	mg/L	ND	

* Determined by regression analysis. ↓ Out of AIJN range due to defect. ↑ Out of AIJN range due to excess.

## Data Availability

All data presented in this study are available upon request from the corresponding authors. All NMR acquisition and preprocessing, as well as statistical outliers, will be publicly available in national NMR repositories, currently under progress.

## Supplementary Materials

The following are available online. Figure S1: ^1^H NMR nutation spectra for the calibration of the 360° pulse length (usec) at an amplitude power level of 23.3 kHz (24 watts) in order to accurately predict the effective 90^°^ pulse length needed for quantitative NMR measurements of the %alcohol content and %alcohol reduction of Cabernet Sauvignon wines with different large-scale fermentation schemes. Figure S2: Refocusing Band-Selective Uniform Response Pure-Phase (REBURP) excitation profile (red) from an 8.35-ppm frequency offset of the shaped pulse optimized for a selective excitation bandwidth of ± 1680 Hz (a total of 3360 Hz irradiation bandwidth from 5.52 ppm to 11.02 ppm) that promotes a selective refocusing of the aromatic chemical shift range (5.5–11 ppm, 3360 Hz), with efficient defocusing of the water-to-ethanol hydroalcoholic resonances. Black: The Fourier-Transform (FT) of the Mz magnetization component excited with the REBURP optimized profile. Figure S3. Signal-to-noise ratio (S/N) calculations for q-^1^H-NMR direct polarization experiment (A, S/N = 33.59 obtained with an optimized receiver gain of 1); {^1^H_water_presat_ NMR}-1D single-pulse NOESY spectrum with an off-resonance shaped-pulse for water and ethanol multi-presaturation during both relaxation delay and mixing times (B, S/N = 10.3 obtained with an optimized receiver gain of 40.3); {^1^H_water_presat_ NMR} spectrum with identical conditions as in B but with an additional continuous-wave decoupling module to eliminate intense ^13^C satellites of ethanol signals (C, S/N = 15.23 obtained with an optimized receiver gain of 25.4); and ^1^H-DPFGE NMR experiments (D, S/N = 68.72 obtained with an optimized receiver gain of 203) acquired at equivalent conditions of spectral width (13 ppm), transmitter frequency offset (4.5 ppm), recovery delays (2 s), and the number of transients (64 scans). Figure S4. Expansion of the aliphatic region of the juice automatized {^1^H_water_presat_ NMR} data matrix, whereas the intelligent binning algorithm of ^1^H resonances is highlighted with gray boxes, such as in Figure 4 of the main text, with aromatic ^1^H spin systems. Figure S5: Brix degrees of raw grape juices, as well as Cabernet Sauvignon wines, pH, total acidity (g/L) and free sulfites (mg/L) of large-scale fermentation carried out in 2018 (blue), 2019 (red), and 2020 (green) year of vintages. Table S1. Microbiological and oenological characteristics of the Non-*Saccharomyces Candida zemplinina* (Enantis Ferm) yeast strain used for alcohol content reductions. Table S2. Microbiological and oenological characteristics of a mixed strain containing the *Saccharomyces uvarum* and *Saccharomyces cerevisiae ex ph. r. bayanus* (herein mentioned as *Saccharomyces Bayanus ex uvarum*) yeast strains used for alcohol content reductions.

## Sample Availability

Samples of the compounds are available from the authors.

## References

[1] Pustjens A.M., Weesepoel Y., van Ruth S.M., Leadley C.E. (2016). 1-Food Fraud and Authenticity: Emerging Issues and Future Trends, In Innovation and Future Trends in Food Manufacturing and Supply Chain Technologies.

[2] Sobolev A.P., Thomas F., Donarski J., Ingallina C., Circi S., Marincola F.C., Capitani D., Mannina L. (2019). Use of NMR applications to tackle future food fraud issues. Trends Food. Sci. Technol..

[3] de Moura Ribeiro M.V., Boralle N., Pezza H.R., Pezza L., Toci A.T. (2017). Authenticity of roasted coffee using ^1^H NMR spectroscopy. J. Food Compos. Anal..

[4] Milani M.I., Rossini E.L., Catelani T.A., Pezza L., Toci A.T., Pezza H.R. (2020). Authentication of roasted and ground coffee samples containing multiple adulterants using NMR and a chemometric approach. Food Control.

[5] Gilard V., Balayssac S., Tinaugus A., Martins N., Martino R., Malet-Martino M. (2015). Detection, identification and quantification by ^1^H NMR of adulterants in 150 herbal dietary supplements marketed for improving sexual performance. J. Pharmaceut. Biomed..

[6] Hachem R., Assemat G., Martins N., Balayssac S., Gilard V., Martino R., Malet-Martino M. (2016). Proton NMR for detection, identification and quantification of adulterants in 160 herbal food supplements marketed for weight loss. J. Pharmaceut. Biomed..

[7] Shi T., Zhu M., Chen Y., Yan X., Chen Q., Wu X., Lin J., Xie M. (2018). ^1^H NMR combined with chemometrics for the rapid detection of adulteration in camellia oils. Food Chem..

[8] Bo Y., Feng J., Xu J., Huang Y., Cai H., Cui X., Dong J., Ding S., Chen Z. (2019). High-resolution pure shift NMR spectroscopy offers better metabolite discrimination in food quality analysis. Food Res. Int..

[9] Kuballa T., Hausler T., Okaru A.O., Neufeld M., Abuga K.O., Kibwage I.O., Rehm J., Luy B., Walch S.G., Lachenmeier D.W. (2018). Detection of counterfeit brand spirits using ^1^H NMR fingerprints in comparison to sensory analysis. Food Chem..

[10] Ciepielowski G., Pacholczyk-Sienicka B., Frączek T., Klajman K., Paneth P., Albrecht Ł. (2019). Comparison of quantitative NMR and IRMS for the authentication of ‘Polish Vodka’. J. Sci. Food Agric..

[11] Santos P.M., Pereira-Filho E.R., Colnago L.A. (2016). Detection and quantification of milk adulteration using time domain nuclear magnetic resonance (TD-NMR). Microchem. J..

[12] Li Q., Yu Z., Zhu D., Meng X., Pang X., Liu Y., Frew R., Chen H., Chen G. (2017). The application of NMR-based milk metabolite analysis in milk authenticity identification. J. Sci. Food Agric..

[13] Hong E., Lee S.Y., Jeong J.Y., Park J.M., Kim B.H., Kwon K., Chun H.S. (2017). Modern analytical methods for the detection of food fraud and adulteration by food category. J. Sci. Food Agric..

[14] Schievano E., Sbrizza M., Zuccato V., Piana L., Tessari M. (2020). NMR carbohydrate profile in tracing acacia honey authenticity. Food Chem..

[15] He C., Liu Y., Liu H., Zheng X., Shen G., Feng J. (2020). Compositional identification and authentication of Chinese honeys by ^1^H NMR combined with multivariate analysis. Food Res. Int..

[16] Lachenmeier D.W., Downey G. (2016). Advances in the detection of the adulteration of alcoholic beverages including unrecorded alcohol. Advances in Food Authenticity Testing.

[17] Ok S. (2017). Detection of olive oil adulteration by low-field NMR relaxometry and UV-Vis spectroscopy upon mixing olive oil with various edible oils. Grasas Aceites.

[18] Jović O., Pičuljan K., Hrenar T., Smolić T., Primožič I. (2019). ^1^H NMR adulteration study of hempseed oil with full chemometric approach on large variable data. Chemometr. Intell. Lab..

[19] Amargianitaki M., Spyros A. (2017). NMR-based metabolomics in wine quality control and authentication. Chem. Biol. Technol. Agric..

[20] Solovyev P.A., Fauhl-Hassek C., Riedl J., Esslinger S., Bontempo L., Camin F. (2021). NMR spectroscopy in wine authentication: An official control perspective. Compr. Rev. Sci. Food Saf..

[21] Viskić M., Bandić L.M., Korenika A.M.J., Jeromel A. (2021). NMR in the service of wine differentiation. Foods.

[22] Lin S., Salcido-Keamo S., Hellberg R.S., Everstine K., Sklare S.A. (2021). Fraud in wine and other alcoholic beverages. Food Fraud.

[23] Marchetti L., Pellati F., Benvenuti S., Bertelli D. (2019). Use of ^1^H NMR to detect the percentage of pure fruit juices in blends. Molecules.

[24] Chater J.M., Mathon C., Larive C.K., Merhaut D.J., Tinoco L.W., Mauk P.A., Zhenyu J., Preece J.E. (2019). Juice quality traits, potassium content, and ^1^H NMR derived metabolites of 14 pomegranate cultivars. J. Berry Res..

[25] Jamin E., Thomas F., Webb G.A. (2018). SNIF-NMR applications in an economic context: Fraud detection in food products. Modern Magnetic Resonance.

[26] Fonseca-Aguiñaga R., Gómez-Ruiz H., Miguel-Cruz F., Romero-Cano L.A. (2020). Analytical characterization of tequila (silver class) using stable isotope analyses of C, O and atomic absorption as additional criteria to determine authenticity of beverage. Food Control.

[27] Christodoulou M., Bradley D., Maréchal A., Nganga J., Béteille R., Moens J., Lejeune F., Montanari F. (2015). Study on the mandatory indication of country of origin or place of provenance of unprocessed foods, single ingredient products and ingredients that represent more than 50% of a food. Food Chain Evaluation Consortium for the Directorate General for Health and Food Safety.

[28] Alves Filho E.G., Silva L.M.A., Ribeiro P.R.V., de Brito E.S., Zocolo G.J., Souza-Leão P.C., Marques A.T.B., Quintela A.L., Larsen F.H., Canuto K.M. (2019). ^1^H NMR and LC-MS-based metabolomic approach for evaluation of the seasonality and viticultural practices in wines from São Francisco River Valley, a Brazilian semi-arid region. Food Chem..

[29] Ün İ., Salim O.K. (2018). Analysis of olive oil for authentication and shelf life determination. J. Food Sci. Techol..

[30] Gougeon L., da Costa G., Richard T., Guyon F. (2019). Wine authenticity by quantitative ^1^H NMR versus multitechnique analysis: A case study. Food Anal. Methods.

[31] Lia F., Vella B., Mangion M.Z., Farrugia C. (2020). Application of ^1^H and ^13^C NMR fingerprinting as a tool for the authentication of Maltese extra virgin olive oil. Foods.

[32] López-Aguilar R., Zuleta-Prada H., Hernández-Montes A., Herbert-Pucheta J.E. (2021). Comparative NMR metabolomics profiling between Mexican ancestral & artisanal mezcals and industrialized wines to discriminate geographical origins, agave species or grape varieties and manufacturing processes as a function of their quality attributes. Foods.

[33] Hu B., Cao Y., Zhu J., Xu W., Wu W. (2019). Analysis of metabolites in chardonnay dry white wine with various inactive yeasts by ^1^H NMR spectroscopy combined with pattern recognition analysis. AMB Expr..

[34] Forino M., Gambuti A., Moio L. (2019). NMR-based systematic analysis of bioactive phytochemicals in red wine. First determination of xanthurenic and oleanic acids. Food Chem..

[35] Kubicova L., Hadacek F., Bachmann G., Weckwerth W., Chobot V. (2019). Coordination complex formation and redox properties of kynurenic and xanthurenic acid can affect brain tissue homeodynamics. Antioxidants.

[36] Cao S., Tian X.L., Yu W.X., Zhou L.P., Dong X.L., Favus M.J., Wong M.S. (2018). Oleanolic acid and ursolic acid improve bone properties and calcium balance and modulate vitamin D metabolism in aged female rats. Front. Pharmacol..

[37] Ayeleso T.B., Matumba M.G., Mukwevho E. (2017). Oleanolic acid and its derivatives: Biological activities and therapeutic potential in chronic diseases. Molecules.

[38] Godelmann R., Fang F., Humpfer E., Schütz B., Bansbach M., Schäfer H., Spraul M. (2013). Targeted and nontargeted wine analysis by ^1^H NMR spectroscopy combined with multivariate statistical analysis. Differentiation of important pa-rameters: Grape variety, geographical origin, year of vintage. J. Agric. Food Chem..

[39] Monakhova Y., Godelmann R., Kuballa T., Mushtakova S., Rutledge D. (2015). Independent components analysis to increase efficiency of discriminant analysis methods (FDA and LDA): Application to NMR fingerprinting of wine. Talanta.

[40] Mazzei P., Spaccini R., Francesca N., Moschetti G., Piccolo A. (2013). Metabolomic by ^1^H NMR spectrosocopy differentiates “Fiano di Avellino” white wines obtained with different yeast strains. J. Agric. Food Chem..

[41] Gougeon L., Da Costa G., Le Mao I., Ma W., Teissedre P., Guyon F., Richard T. (2018). Wine analysis and authenticity using ^1^H-NMR metabolomics data: Application to Chinese wines. Food Anal. Methods.

[42] Hu B., Gao J., Xu S., Zhu J., Fan X., Zhou X. (2020). Quality evaluation of different varieties of dry red wine based on nuclear magnetic resonance metabolomics. Appl. Biol. Chem..

[43] Del Fresno J.M., Escott C., Loira I., Herbert-Pucheta J.E., Schneider R., Carrau F., Cuerda R., Morata A. (2020). Impact of *Hanseniaspora vineae* in alcoholic fermentation and ageing on lees of high-quality white wine. Fermentation.

[44] Hwang T.J., Shaka A.J. (1995). Water suppression that works. Excitation sculpting using arbitrary wave-forms and pulsed-field gradients. J. Magn. Reson. A.

[45] Geen H., Freeman R. (1991). Band-selective radiofrequency pulses. J. Magn. Reson..

[46] Herbert-Pucheta J.E., Pino-Villar C., Rodríguez-González F., Padilla-Maya G., Milmo-Brittingham D., Zepeda-Vallejo L.G. (2019). “One-shot” analysis of wine parameters in non-*Saccharomyces* large-scale alcohol reduction processes with one- and two-dimensional nuclear magnetic resonance. BIO Web Conf..

[47] Herbert-Pucheta J.E., López-Morales C.A., Medina-Rivero E., Estrada-Parra S., Pérez-Tapia S.M., Zepeda-Vallejo L.G. (2021). Consistency of a dialyzable leucocyte extract manufactured at GMP facilities by nuclear magnetic resonance spectroscopy. Pharm. Biomed. Anal..

[48] Spraul M., Schütz B., Humpfer E., Mörtter M., Schäfer H., Koswig S., Rinke P. (2009). Mixture analysis by NMR as applied to fruit juice quality control. Magn. Reson. Chem..

[49] The AIJN Code of Practice. https://aijn.eu/en/the-aijn-code-of-practice.

[50] Pérez-Enciso M., Tenenhaus M. (2003). Prediction of clinical outcome with microarray data: A partial least squares discriminant analysis (PLS-DA) approach. Hum. Genet..

[51] Lê Cao K.A., Boitard S., Besse P. (2011). Sparse PLS discriminant analysis: Biologically relevant feature se-lection and graphical displays for multiclass problems. BMC Bioinform..

[52] Worley B., Powers R. (2013). Multivariate analysis in metabolomics. Curr. Metabol..

[53] Spraul M., Schütz B., Rinke P., Koswig S., Humpfer E., Schäfer H., Mörtter M., Fang F., Marx U.C., Minoja A. (2009). NMR-based multi parametric quality control of fruit juices: SGF profiling. Nutrients.

[54] Schmidtke L.M., Blackman J.W. (2012). Production technologies for reduced alcoholic wines. J. Food Sci..

[55] Contreras A., Hidalgo C., Schmidt S., Henschke P.A., Curtin C., Varela C. (2015). The application of non-*Saccharomyces* yeast in fermentations with limited aeration as a strategy for the production of wine with reduced alcohol content. Int. J. Food Microbiol..

[56] Obbi M., De Vero L., Solieri L., Comitini F., Oro L., Giudici P., Ciani M. (2014). Fermentative aptitude of non-*Saccharomyces* wine yeast for reduction in the ethanol content in wine. Eur. Food Res. Technol..

[57] Del Fresno J.M., Morata A., Loira I., Bañuelos M.A., Escott C., Benito S., Chamorro C.G., Suárez-Lepe J.A. (2017). Use of non-*Saccharomyces* in single-culture, mixed and sequential fermentation to improve red wine quality. Eur. Food Res. Technol..

[58] Benito A., Hofmann T., Laier M., Lochbüler B., Schüttler A., Ebert K., Fritsch S., Röcker J., Rauhut D. (2015). Effect on quality and composition of Riesling wines fermented by sequential inoculation with non-*Saccharomyces* and *Saccharomyces cerevisiae*. Eur. Food Res. Technol..

[59] Varela C., Barker A., Tran T., Borneman A., Curtin C. (2017). Sensory profile and volatile aroma composition of reduced alcohol Merlot wines fermented with *Metschnikowia pulcherrima* and *Saccharomyces uvarum*. Int. J. Food Microbiol..

[60] Gobbi M., Comitini F., Domizio P., Romani C., Lencioni L., Mannazzu I., Ciani M. (2013). *Lachancea thermotolerans* and *Saccharomyces cerevisiae* in simultaneous and sequential co-fermentation: A strategy to enhance acidity and improve the overall quality of wine. Food Microbiol..

[61] de Meyer T., Sinnaeve D., van Gasse B., Tsiporkova E., Rietzschel E., de Buyzere M., Gillebert T., Bekaert S., Martins J., van Criekinge W. (2008). NMR-based characterization of metabolic alterations in hypertension using an adaptive, intelligent binning algorithm. Anal. Chem..

[62] Shishmarev D., Otting G. (2011). Radiation damping on cryoprobes. J. Magn. Reason..

[63] Ebrahimi P., Viereck N., Bro R., Engelsen S.B., Webb G.A. (2018). Chemometric Analysis of NMR Spectra. Modern Magnetic Resonance.

[64] Jacob D., Deborde C., Lefevbre M., Maucourt M., Moing A. (2017). NMRProcFlow: A graphical and interactive tool dedicated to 1D spectra processing for NMR-based metabolomics. Metabolomics.

[65] Chong J., Wishart D.S., Xia J. (2019). Using MetaboAnalyst 4.0 for comprehensive and integrative metabolomics data analysis. Curr. Protoc. Bioinform..

[66] Holmes E., Loo R.L., Stamler J., Bictash M., Yap I.K.S., Chan Q., Ebbels T., De Iorio M., Brown I.J., Veselkov K.A. (2008). Human metabolic phenotype diversity and its association with diet and blood pressure. Nature.

[67] Xu Q.-S., Liang Y.-Z. (2001). Monte Carlo cross validation. Chemom. Intell. Lab. Syst..

