# Supplementary material for: Nuclear Magnetic Resonance Metabolomics with Double Pulsed-Field-Gradient Echo and Automatized Solvent Suppression Spectroscopy for Multivariate Data Matrix Applied in Novel Wine and Juice Discriminant Analysis
Source: Molecules. 2021 Jul 7;26(14):4146. doi: 10.3390/molecules26144146 (PMC8307358; doi:10.3390/molecules26144146)
Supplement: Supplementary file 1 [file molecules-26-04146-s001.zip › molecules-1257654-supplementary.pdf]

molecules

Article

Nuclear Magnetic Resonance Metabolomics with Double Pulsed-Field-Gradient Echo and Automatized Solvent Suppression Spectroscopy for Multivariate Data Matrix Applied in Novel Wine and Juice Discriminant Analysis

José Enrique Herbert-Pucheta[1,2], José Daniel Lozada-Ramírez[3], Ana E. Ortega-Regules[4], Luis Ricardo Hernández[3,*] and Cecilia Anaya de Parrodi[3,*]

1 Consejo Nacional de Ciencia y Tecnología-Laboratorio Nacional de Investigación y Servicio Agroalimentario y Forestal, Universidad Autónoma Chapingo. Carretera México-Texcoco km 38.5, Chapingo, Estado de México 56230, Mexico; jherbertp@ipn.mx
2 Departamento de Química Orgánica, Escuela Nacional de Ciencias Biológicas, Instituto Politécnico Nacional. Prolongación de Carpio y Plan de Ayala s/n, Colonia Santo Tomás, Ciudad de México 11340, Mexico
3 Departamento de Ciencias Químico Biológicas, Universidad de las Américas Puebla, San Andrés Cholula 72810, Mexico; jose.lozada@udlap.mx
4 Departamento de Ciencias de la Salud, Universidad de las Américas Puebla, San Andrés Cholula 72810, Mexico; ana.ortega@udlap.mx
* Correspondence: luisr.hernandez@udlap.mx ((L.R.H.); cecilia.anaya@udlap.mx (C. A. de P.); Tel.: +52-222-2292412 (L.R.H.); +52-222-2292005 (C.A. de P.)

Supporting Information Material

Citation: Herbert-Pucheta, J. E.; Lozada-Ramírez, J. D.; Ortega-Regules, A. E.; Hernández, L. R.; Anaya de Parrodi, C. Nuclear Magnetic Resonance Metabolomics with Double Pulsed Field Gradient Echo and Automatized Solvent Suppression Spectroscopy for Multivariate Data Matrix Applied in Novel Wine and Juice Discriminant Analysis. *Molecules* **2021**, *26*, x. https://doi.org/10.3390/xxxxx

Academic Editor: Alessandra Biancolillo; Angelo Antonio D'Archivio

Received: 27 May 2021
Accepted: 23 June 2021
Published: date

Publisher's Note: MDPI stays neutral with regard to jurisdictional claims in published maps and institutional affiliations.

Copyright: © 2021 by the authors. Submitted for possible open access publication under the terms and conditions of the Creative Commons Attribution (CC BY) license (https://creativecommons.org/licenses/by/4.0/).

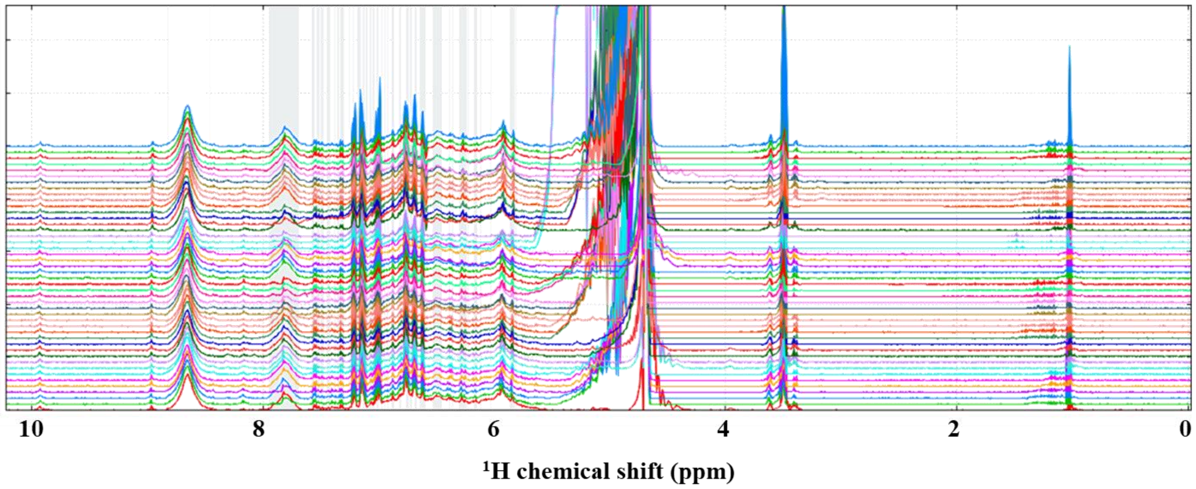

Figure S1. Stacked one-dimensional DPFGE proton NMR spectra of Cabernet Sauvignon wines, fermented with *Saccharomyces cerevisiae*, co-inoculation with *Candida zemplinina*, and inoculation with *Saccharomyces bayanus ex uvarum* (see Materials and methods). Pure in-phase selective excitation of the 5.5–11 ppm chemical shift range with DPFGE was applied for all wine samples at identical conditions for obtaining effective frequency selection without phase distortions within the full excitation bandwidth, despite the residual 4.7, 3.47, and 1.005 ppm water-to-ethanol observed resonances (see Materials and Methods). Gray boxes highlight the binning strategy to obtain data dimensionality.

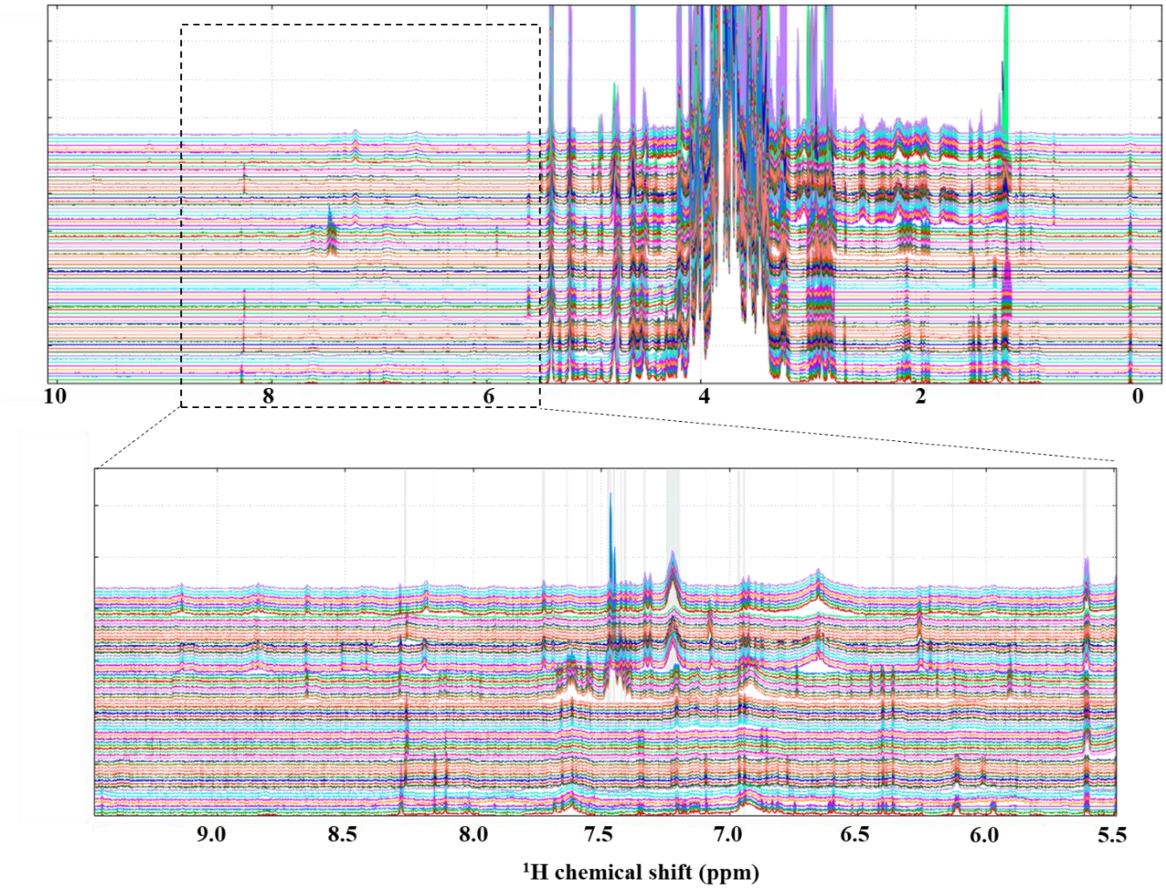

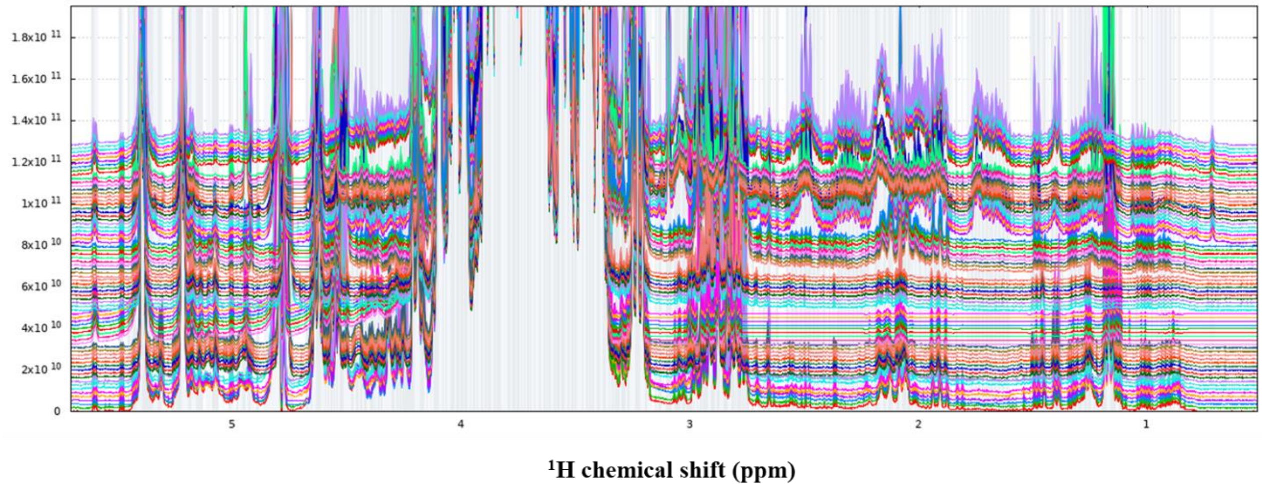

Figure S2. Stacked one-dimensional {$^{1}H_{water\_presat}$ NMR} spectra of apple, orange and apricot 100% juices in different presentations. a) Made from concentrate. b) Not-from concentrate (commercial, see Table 1). c) Nectars. d) Purees. Top: Full ^{1}H frequency range; middle: aromatic ^{1}H frequency range expansion from 5.5 to 9.3 ppm and bottom: Expansion of the aliphatic region of Juices' automatized {$^{1}H_{water\_presat}$ NMR} data matrix. The intelligent binning algorithm of ^{1}H resonances for obtaining data dimensionality in both expansions is highlighted with gray boxes.

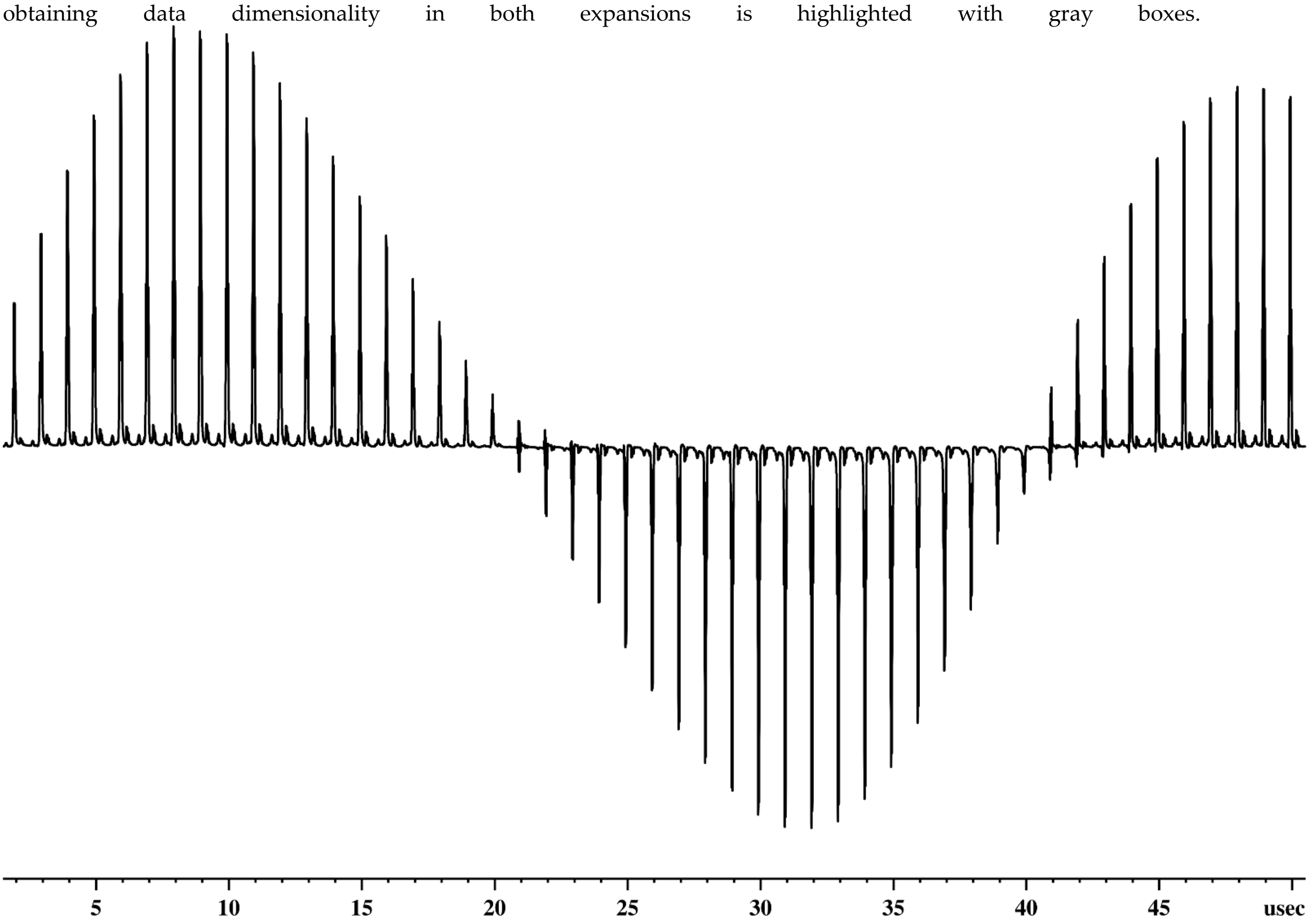

.

Figure S3. ^{1}H NMR nutation spectra for calibration of 360° pulse length (usec) at an amplitude power level of 23.3 kHz (24 Watts) in order to accurately predict the effective 90° pulse length needed for quantitative NMR measurements of %alcohol content and %alcohol reduction of Cabernet Sauvignon wines with different large-scale fermentation schemes (Figure 1, Main text).

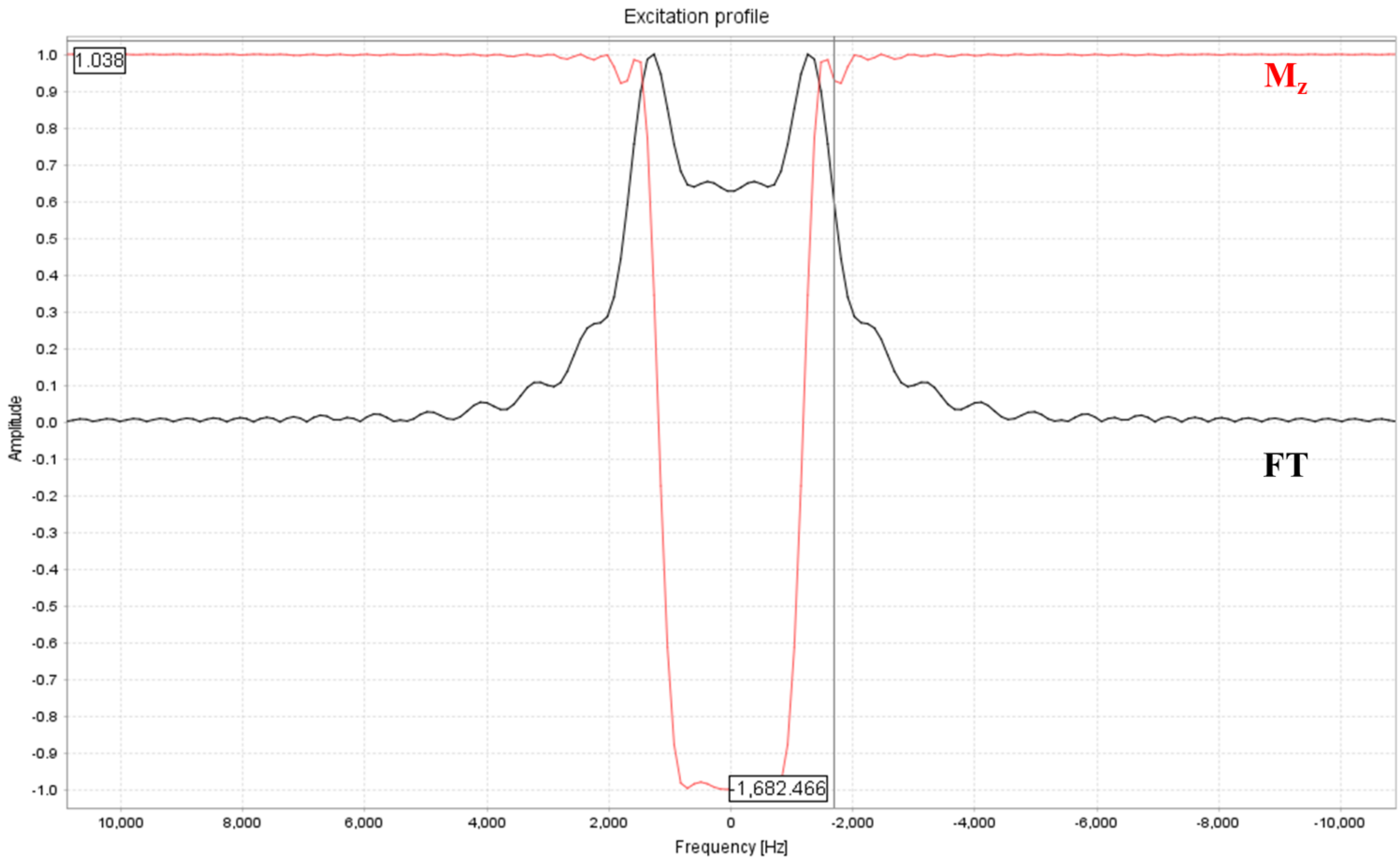

Figure S4. Refocusing Band Selective Uniform-Response Pure-Phase (REBURP) excitation profile (red) from an 8.35 ppm frequency offset of the shaped pulse, optimized for a selective excitation bandwidth of ± 1680 Hz (a total of 3360 Hz irradiation bandwidth from 5.52 ppm to 11.02 ppm) that promotes a selective refocusing of the aromatic chemical shift range (5.5–11 ppm, 3360 Hz) with efficient defocusing of the water-to-ethanol hydroalcoholic resonances. Black: The Fourier Transform (FT) of the Mz magnetization component excited with the REBURP optimized profile.

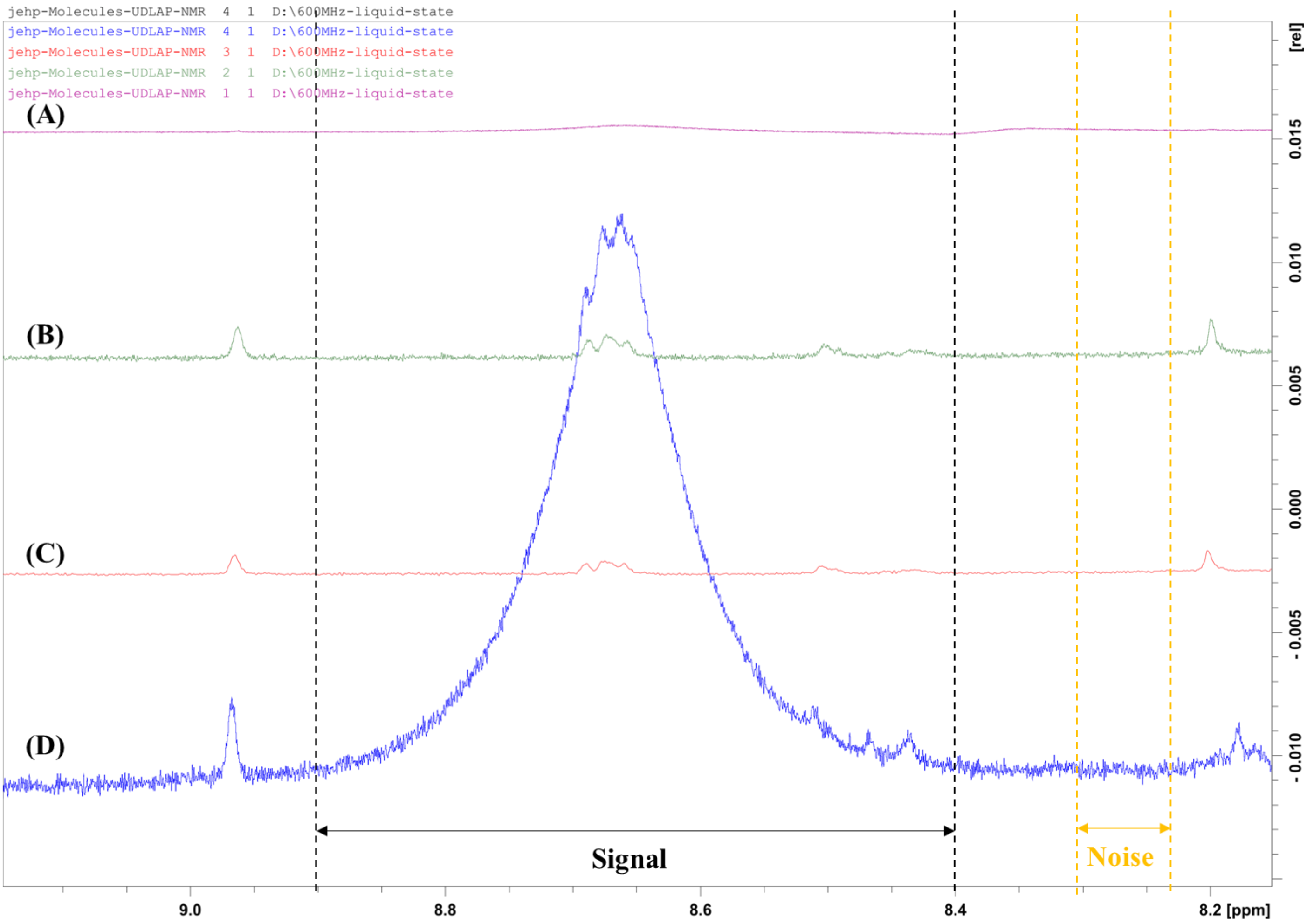

Figure S5. Signal-to-noise ratio (S/N) calculations for q-^{1}H- NMR direct polarization experiment (A, S/N= 33.59 obtained with an optimized receiver gain of 1), {$^{1}H_{water_presat}$ NMR} -1D single-pulse NOESY spectrum with an off-resonance shaped-pulse for water and ethanol multipresaturation during both relaxation delay and mixing times (B, S/N= 10.3 obtained with an optimized receiver gain of 40.3), {$^{1}H_{water\_presat}$ NMR} spectrum with identical conditions as in B but with an additional continuous-wave decoupling module to eliminate intense ^{13}C satellites of ethanol signals (C, S/N= 15.23 obtained with an optimized receiver gain of 25.4) and ^{1}H-DPFGE NMR experiments (D, S/N= 68.72 obtained with an optimized receiver gain of 203) acquired at equivalent conditions of spectral width (13 ppm), transmitter frequency offset (4.5 ppm), recovery delays (2 seconds), and number of transients (64 scans).

Table S1. Microbiological and oenological characteristics of Non-*Saccharomyces Candida zemplinina* (Enantis Ferm) yeast strain, used for alcohol content reductions.

Candida Zemplinina	
Fermentation temperature	15–26 °C
Fermentation velocity	Slow
Alcohol tolerance	≤ 10% *v*/*v*
SO_2 resistance	< 20 ppm free
Volatile acidity (VA) production	20–30% less than *S. cerevisiae*
Glycerol production	Very high
H_2S production	Very low
SO_2 production	Very low

Nitrogen need	Not Specified

Table S2. Microbiological and oenological characteristics of mixed strain containing *Saccharomyces uvarum* and *Saccharomyces cerevisiae ex ph. r. bayanus* (herein mentioned as *Saccharomyces Bayanus ex uvarum*) yeast strain, used for alcohol content reductions.

S. Bayanus ex uvarum	
Fermentation temperature	15–20 °C
Fermentation velocity	Moderate
Alcohol tolerance	≤ 16% *v/v*
SO_2 resistance	High
Volatile acidity (VA) production	Very low (< 0.25 g/L)
Glycerol production	Very high
H_2S production	Medium
SO_2 production	Low
Nitrogen need	150g/hL of YAN

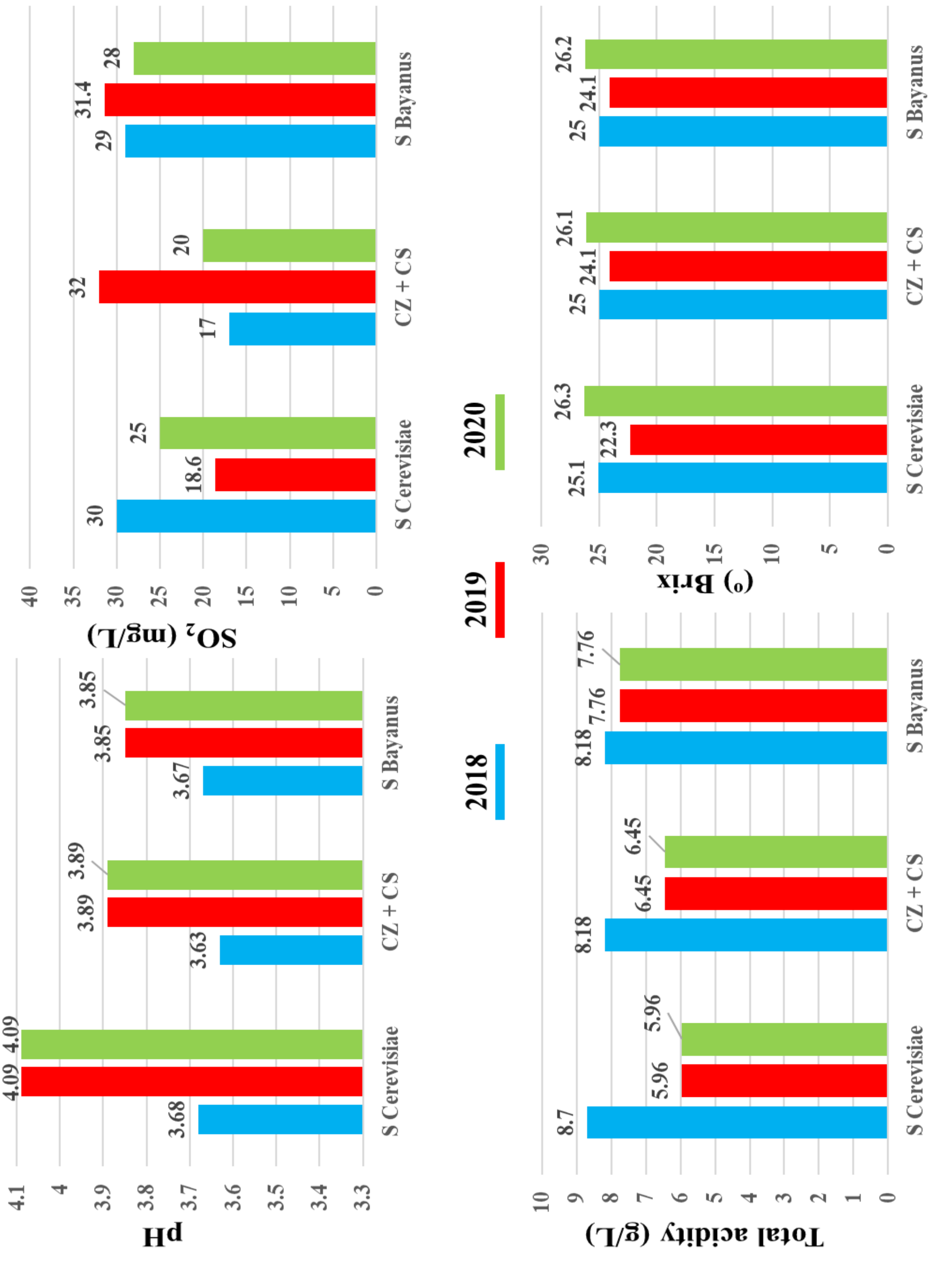

Figure S6. Degrees Brix of raw grape juices as well as Cabernet Sauvignon wines' pH, total acidity (g/L), and free sulfites (mg/L) of large-scale fermentation carried out in 2018 (blue), 2019 (red), and 2020 (green) year of vintages.
